# FLT3L combined with GM-CSF induced dendritic cells drive broad tumor-specific CD8^+^ T cell responses and remodel the tumor microenvironment to enhance anti-tumor efficacy

**DOI:** 10.3389/fimmu.2025.1649891

**Published:** 2025-09-04

**Authors:** Qian Zheng, Jiajie Zhang, He Sui, Yu Sun, Ningning Lv, Lin Liu, Ming Qu, Jiateng Tan, Bin Zhang, Zhanhao Mo

**Affiliations:** ^1^ Department of Endoscopy Center, China-Japan Union Hospital of Jilin University, Changchun, China; ^2^ Department of Research & Development, Deer-spring Medtech Inc., Suzhou, China; ^3^ Department of Radiology, China-Japan Union Hospital of Jilin University, Changchun, China; ^4^ Department of Interventional Department, China-Japan Union Hospital of Jilin University, Changchun, China; ^5^ Department of Ultrasound, China-Japan Union Hospital of Jilin University, Changchun, China

**Keywords:** dendritic cells, DCs, FLT3L, GM-CSF, cDC1, anti-tumor efficacy

## Abstract

**Background:**

Dendritic cells (DCs) play a crucial role in anti-tumor immunity by capturing, processing, and presenting tumor antigens to T cells, making DC-based immunotherapy a promising approach for cancer treatment. However, the most commonly used clinical strategy still relies on inducing DCs *in vitro* using granulocyte-macrophage colony-stimulating factor (GM-CSF) and interleukin-4 (IL - 4) (GM/IL4-DCs), which often results in a heterogeneous cell population with suboptimal anti-tumor function. Here, we compared DCs generated by co-stimulating with FMS-like tyrosine kinase 3 ligand (FLT3L) and GM-CSF (FL/GM-DCs) with the conventional GM/IL4-DCs.

**Method:**

To compare the functional differences of DCs induced by different methods, we conducted a comprehensive study. Mouse bone marrow cells were continuously cultured for 9 days in a FLT3L/GM-CSF-containing medium. After cell collection, we analyzed the composition, subpopulations, and status of FL/GM-DCs using flow cytometry and scRNA-seq. Flow cytometry was also used to assess their antigen presentation and ability to stimulate T cells. *In vivo* experiments were performed to examine their distribution, anti-tumor effects, and therapeutic responses in tumor models. Finally, combining scRNA-seq and scTCR-seq, we explored the mechanisms by which FL/GM-DCs reshape the tumor microenvironment.

**Results:**

The results showed that FL/GM-DCs exhibited a unique subpopulation distribution, characterized by an abundance of conventional cDC subpopulations, and demonstrated enhanced cross-antigen presentation capabilities. Notably, FL/GM-DCs were able to induce a broader and more tumor-specific CD8^+^ T cell response, effectively reshaping the tumor microenvironment by promoting the infiltration of cytotoxic T lymphocytes (CTLs) and reducing immunosuppressive components. In contrast, GM/IL4-DCs contained fewer cDC subpopulations, eliciting a weaker initial CD8^+^ T cell response and yielding relatively inferior anti-tumor effects.

**Conclusion:**

In summary, FLT3L combined with GM-CSF induced DCs, through their unique subpopulation composition and functional state, can more effectively expand tumor-specific CD8^+^ T cells and reshape the tumor microenvironment, thereby achieving superior immunotherapy outcomes. This study highlights the potential of FL/GM-DCs as a next-generation DC platform, paving the way for improved clinical translation of DC-based adoptive cancer immunotherapies.

## Introduction

Dendritic cells (DCs) are essential for orchestrating anti-tumor immune responses (1). As specialized antigen-presenting cells, they efficiently capture, process, and present tumor antigens (2, 3). Upon activation, DCs secrete a wide array of cytokines that influence immune effector cell chemotaxis, activation, and proliferation. Past studies have highlighted the importance of colocalization and intercellular communication (cross-talk) between DCs and effector cells within the tumor microenvironment for maximizing the cytotoxic activity of effector cells against tumor cells (4–6). These characteristics make adoptive DC therapy a promising approach in cancer treatment (7–10). More than a decade ago, the United States Food and Drug Administration approved Provenge, the first DC-based therapy, for advanced prostate cancer (11). Since then, DC therapies have been applied to a growing range of malignancies, including melanoma (12), glioma (13), and hepatocellular carcinoma (14). Despite these advances, clinical applications and preclinical studies have shown that the therapeutic efficacy of DC therapies remains modest.

DCs encompass a diverse array of subtypes, including plasmacytoid DCs (pDCs), conventional DCs (cDCs), monocyte-derived DCs (moDCs), and the recently identified DC3 subtype (15–18). cDCs—particularly the cDC1 subset—are renowned for their potent anti-tumor properties, especially their ability to activate cytotoxic CD8^+^ T cells. Clinical evidence suggests that higher densities of cDC1 within the tumor microenvironment correlate with improved patient outcomes across multiple tumor types (6, 19, 20). In contrast, cDC2 cells exhibit greater heterogeneity compared to cDC1, and their prognostic significance remains less clearly defined (21). Emerging research indicates that cDC2 can effectively present antigens to both CD4^+^ and CD8^+^ T cells, particularly adept at priming Th1 responses (22). Preliminary findings from clinical trials involving cDC-based tumor therapies have further demonstrated their potential in suppressing tumor progression, highlighting their promise in the field of cancer immunotherapy.

The methods used for generating DCs *in vitro* significantly influence their phenotypic characteristics and functional states. Currently, the most widely used strategy employs granulocyte-macrophage colony-stimulating factor (GM-CSF) and interleukin-4 (IL - 4) to induce DC differentiation from progenitor cells or peripheral monocytes (23, 24). Despite advances using this method, clinical outcomes have remained modest (25). A critical limitation is that GM-CSF combined IL - 4 culture systems produce highly heterogeneous cell populations, comprising not only DCs but also substantial fractions of monocytic cells including macrophages (26). Although macrophages generated under these conditions can efficiently present antigens, they often exhibit limited immunostimulatory capacities compared with bona fide DCs and, due to their high plasticity, can readily shift toward immunosuppressive phenotypes in the tumor microenvironment (27, 28).

The FLT3 ligand (FLT3L)-based differentiation pathway has emerged as a promising alternative, given its crucial role in the natural development and expansion of DC subsets *in vivo* (29–31). Clinical studies utilizing FLT3L have also shown significant increases in patient DC counts and demonstrated potential therapeutic benefits (32). By mimicking physiological DC development, FLT3L-based methods yield a population of DCs closely resembling naturally occurring subsets, with greater potential for clinical translation. However, detailed characterization of DC subpopulations, their maturation states, and the precise mechanisms by which these DCs reshape the tumor microenvironment upon adoptive transfer remain largely unexplored.

In this study, we performed comprehensive immunophenotyping of DCs generated via FLT3L/GM-CSF-based induction (FL/GM-DCs), characterizing their global immune populations, DC subsets, and activation states. This study represented the first direct comparison of FLT3L+GM-CSF and GM-CSF+IL-4-derived DC cultures using single-cell RNA sequencing (scRNA-seq), enabling an unprecedented resolution of DC subset heterogeneity and uncovering phenotypically distinct clusters with potential antitumor relevance. Through *in vitro* experiments, we assessed the antigen-presenting capacity of these FL/GM-DCs and their ability to stimulate T cell activation and proliferation. To elucidate the underlying mechanisms, we innovatively combined DC therapy with single-cell T cell receptor sequencing (scTCR-seq), allowing us to track tumor-specific CD8^+^ T cell expansion *in vivo*. Directly comparing these parameters with DCs generated by the conventional GM-CSF/IL-4-based method (GM/IL4-DCs), our findings demonstrate that FL/GM-DCs effectively reshape the tumor microenvironment, contributing to long-term immune remodeling and functional persistence. These findings provide critical insights into the superior therapeutic potential and mechanistic advantages of FL/GM-DCs, offering strong support for their clinical translation in DC-based cancer immunotherapy.

## Materials and methods

### Cell lines

MC38 colorectal carcinoma cells (MC38, RRID: CVCL_B288) were obtained from Cyagen Biosciences; B16F10 melanoma cells (B16, RRID: CVCL_0159) and a B16F10 variant expressing ovalbumin (OVA, B16-OVA, RRID: CVCL_WM78) were acquired from MeisenCTCC. Cells were cultured at 37 °C with 5% CO_2_ in Dulbecco’s Modified Eagle Medium (Cytiva) supplemented with 10% fetal bovine serum (Absin) and 1% penicillin-streptomycin (Coolaber). When cells reached confluence in culture flasks, they were passaged using 0.25% trypsin (Cytiva) containing ethylenediaminetetraacetic acid (EDTA). For each cell line, the number of passages was strictly controlled and did not exceed 10. Mycoplasma contamination testing in our laboratory confirmed that all cell lines were free of contamination.

### Mice

All studies were conducted using C57BL/6J mice. C57BL/6J wild-type mice (RRID: IMSR_JAX:000664) were purchased from Beijing Vital River Laboratory Animal Technology Company; B6.SJL-Ptprca Pepc/BoyJ (B6 CD45.1, RRID: IMSR_JAX:002014) mouse is a variant of the C57BL/6J strain that expresses the CD45.1 (Ptprca) allele, and these mice were purchased from Cyagen Biosciences. C57BL/6-Tg(TcraTcrb)1100Mjb/J (OT-I TCR Transgenic Mouse, RRID: IMSR_JAX:003831) and B6.CG-TG(TcraTcrb)425Cbn/J (OT-II TCR Transgenic Mouse, RRID: IMSR_JAX:004194) mice were also obtained from Cyagen Biosciences. Mice were maintained under specific pathogen-free (SPF) conditions at Wish Technology. Animals used in the experiments were 4 – 6 weeks old, sex-matched, and randomized into groups of up to five mice per cage. Housing conditions were maintained at 18 – 24°C with 40 – 60% humidity. All animal experiments were approved by the Animal Experimentation Ethics Committee of Jilin University (#SYXK-2023-0010). All procedures followed institutional guidelines and complied with national regulations on animal welfare.

### Isolation of mouse bone marrow cells

Femurs and tibias were harvested from 4 – 6-week-old C57BL/6J wild-type mice; skin and muscle tissue were carefully removed. Both ends of each bone were cut, and bone marrow was flushed out using a 1 mL syringe filled with phosphate-buffered saline (Biosharp). The bone marrow cavity was rinsed repeatedly until it appeared completely white. The bone marrow suspension was filtered through a 40-μm cell strainer (BD Biosciences) and centrifuged at 250 × g for 8 min to pellet the cells. Red blood cells were lysed using red blood cell lysis buffer (Solarbio) for 6 min. After 2 – 3 washes, approximately 10^7^ cells were seeded in a T75 cell culture flask.

### Generation of mouse FL/GM-DCs

Mouse bone marrow cells were cultured in RPMI 1640 complete medium (Cytiva) supplemented with 100 ng/mL FLT3L (SinoBiological) and 10 ng/mL GM-CSF (SinoBiological). The medium was replaced every 3 days. On day 9, tumor tissue lysate was added to the culture medium for overnight antigen loading. The following day, the original culture medium was removed and replaced with RPMI 1640 complete medium containing 5μg/mL R848 (Absin) and 10μg/mL poly I (Absin). Cells were incubated for an additional 4 – 6h. At the end of the incubation, both loosely adherent and suspension cells were harvested for subsequent experiments.

### Generation of mouse GM/IL4-DCs

To generate GM/IL4-DCs, mouse bone marrow cells were cultured in RPMI 1640 medium containing 50ng/mL GM-CSF (SinoBiological) and 50ng/mL IL - 4 (SinoBiological). The medium was replaced every 3 days. On day 7, tumor tissue lysate was added to the culture medium for overnight antigen loading. The following day, the culture medium was replaced with RPMI 1640 complete medium containing 5μg/mL R848 and 10μg/mL poly I for 4 – 6h. At the end of the incubation, both loosely adherent and suspension cells were harvested for subsequent experiments.

### Tumor lysate preparation

Tumor cells in logarithmic phase were resuspended in PBS, counted, and subcutaneously injected into the right flank of 4- to 6-week-old C57BL/6J mice. When tumors reached an average volume of 1000mm^3^, mice were euthanized, and tumors were excised, rinsed with cold PBS, and weighed. Tumor tissue (5g) was minced, digested in 5mL of digestion buffer at 37°C for 20min, and filtered through a 40μm mesh. The filtrate was centrifuged at 1200rpm for 10min, and the cell pellet was resuspended in FBS-free RPMI 1640 medium to a concentration of 1×10^7^/mL. The suspension underwent five freeze-thaw cycles (-80°C/37°C, 15min each) and sonication (200W, 3s on/3s off, 5min). After centrifugation at 12,000rpm for 5min at 4°C, the supernatant was collected, quantified using a BCA assay, and stored at -80°C. For specific applications, B16-OVA tumor lysates were used in OT-I/II T cell proliferation assays, B16F10 lysates were applied in *in vitro* functional experiments, and both MC38 and B16F10 lysates were utilized in *in vivo* antitumor studies. The final protein concentration was determined to be 5mg/mL.

### Antigen phagocytosis and presentation assay

DCs were generated from mouse bone marrow cells as described above and incubated in six-well plates (2 × 10^6^ cells per well) with 1μg OVA–fluorescein isothiocyanate (FITC; Solarbio) at 37°C for 4 – 6 h, protected from light. Cells were washed twice with autoMACS Running Buffer (Miltenyi Biotech) and analyzed by flow cytometry. For flow cytometry analysis, DCs were first gated according to MHCII and CD11c expression, followed by evaluation of their phagocytic capacity.

For antigen presentation, DCs were seeded in six-well plates at a density of 2 × 10^6^ cells per well. Each well was received separately 1 ug/ml, 10 ug/ml, 100 ug/ml, 500ug/ml, and 1 mg/mL OVA solution (Yuanye Bio) and was incubated at 37 °C for 24 h. After two washes, cells were stained with OVA257 - 264 (SIINFEKL) peptide bound to H - 2Kb Monoclonal Antibody-APC (Thermo Fisher, Cat# 17 - 5743-80, eBio25-D1.16 (25-D1.16), RRID: AB_1311288) and then analyzed by flow cytometry to assess the level of antigen presentation.

### OT-I/II T cell proliferation assay

Single-cell suspensions were prepared from OT-I or OT-II TCR Transgenic Mouse spleen, and red blood cells were removed. OT-I CD8^+^T cells or OT-II CD4^+^T cells were isolated using naïve CD8^+^T cell or CD4^+^T cell isolation kits (Miltenyi Biotech) and labeled with carboxyfluorescein diacetate succinimidyl ester (CFSE; Solarbio). DCs were cultured as described above; 3 × 10^5^ cells per well were plated and pulsed with B16-OVA tumor lysate for 4 – 6 h. Antigen-loaded DCs were washed and co-cultured with 2 × 10^6^ OT-I or OT-II cells in six-well plates. Co-cultures were maintained for 96 h. T cell activation and proliferation were evaluated by staining cells with CFSE and CD44 Monoclonal Antibody-Super Bright™436 (Thermo Fisher, Cat# 62 - 0441-82, IM7, RRID: AB_2662589), then analyzing them via flow cytometry.

### Adoptive DC transfer assay

For adoptive transfer to tumor-free mice, DCs were generated from B6 CD45.1 mouse bone marrow cells. C57BL/6J wild-type mice received 1 × 10^6^ CD45.1^+^ DCs and 1 × 10^6^ CD45.1^+^ T cells via footpad injection. Recipient mice were sacrificed 7- or 14-days post-transfer; their lymph nodes were harvested for analysis of donor-derived cells. The extracted cells were stained with CD45.1 Monoclonal Antibody (FITC, Biolegend, Cat#110705, A20, RRID: AB_313494), followed by the establishment of a gating strategy in flow cytometry to isolate the CD45.1^+^ cells. Subsequently, CD3 Monoclonal Antibody (APC, Elabscience, Cat#E-AB-F1013E, 17A2, RRID: AB_3675272) and CD8a Monoclonal Antibody (FITC, Thermo Fisher, Cat# 11 - 0081-82, 53 - 6.7, RRID: AB_464915) were used to further label the CD8^+^ T cells, and the percentage changes of CD8^+^ T cells at different time points, such as day 7 and day 14, were calculated.

For adoptive transfer to tumor-bearing mice, DCs were also prepared from B6 CD45.1 mouse bone marrow cells. C57BL/6J wild-type mice were subcutaneously injected with 8 × 10^5^ MC38 tumor cells in the right flank. Three days after tumor cell injection, these mice were treated with DCs via footpad injection. Each mouse received 2.5 × 10^6^ CD45.1^+^ DCs twice per week for the first 2 weeks and once per week for the subsequent 2 weeks. Recipient mice were sacrificed 7, 14, or 30 days after the initial DC transfer; their lymph nodes and tumors were collected to assess donor-derived cells. Using flow cytometry, we analyzed the proportion changes of CD45.1^+^ cells in lymph nodes and tumor tissues at different time points. Furthermore, based on this, we explored the specific role of CD45.1^+^ cells in the anti-tumor immune response through MHC Class II (I-A/I-E) Monoclonal Antibody-FITC (Thermo Fisher, Cat#11-5321-82, M5/114.15.2, RRID: AB_465232), CD86 Monoclonal Antibody-APC (Biolegend, Cat#105011, GL1, RRID: AB_493343), and XCR1 Monoclonal Antibody-Brilliant Violet 785™ (Biolegend, Cat#148225, ZET, RRID: AB_ 2783119), staining.

### Flow cytometry

Single-cell suspensions were prepared before staining. To ensure high cell viability, dead cells were removed using the Dead Cell Removal Kit (Miltenyi Biotec, Cat#130-090-101), following the manufacturer’s instructions. The flow-through containing viable cells was collected, and cell viability was assessed by trypan blue exclusion assay using a hemocytometer. Only samples with viability consistently above 95% were used for subsequent staining and flow cytometry analysis. Then cell-surface Fc receptors were blocked using TruStain FcX™ PLUS Antiboy (Biolegend, Cat# 156604, S17011E, RRID: AB_2783138) for 20 min at 4°C. Primary antibodies, mixed at the recommended concentrations, were added to the cell suspensions and gently vortexed. Cells were incubated with the antibody mixture for 30 min at 4°C. Flow cytometry was performed using the FongCyte™ instrument (Challenbio), and data were analyzed with FlowJo v10.1 (RRID:SCR_008520). To set up fluorescence compensation for flow cytometry, prepare cell samples identical to the experimental group and set up single-stained control tubes for each fluorescence channel. Each tube should contain only one fluorescently labeled antibody. The tubes are then stained, washed, and fixed under the same conditions as the experimental samples. Subsequently, collect data from each single-stained tube using a flow cytometer, and set up the compensation matrix using analysis software to ensure accurate correction of spectral overlap between fluorescence signals, thereby guaranteeing the accuracy of multicolor experimental data.

Antibodies targeting the following proteins were used:

MHC Class II (I-A/I-E) Monoclonal Antibody-FITC (Thermo Fisher, Cat#11-5321-82, M5/114.15.2, RRID: AB_465232), CD11c Monoclonal Antibody-eFluor™ 450 (Thermo Fisher, Cat#48-0114-82, N418, RRID: AB_1548654), XCR1 Monoclonal Antibody-PE/Cyanine7 (Biolegend, Cat#148237, ZET, RRID: AB_3106079), DCIR2 Monoclonal Antibody-PerCP-eFluor™ 710 (Thermo Fisher, Cat#46-5884-80, 33D1, RRID: AB_2573790), CD172a (SIRP alpha) Monoclonal Antibody-PE (Thermo Fisher, Cat#12-1721-82, P84, RRID: AB_11151506), CD103 (Integrin alpha E) Monoclonal Antibody-Brilliant Violet™ 711 (Thermo Fisher, Cat#407-1031-82, 2E7, RRID: AB_2942156), CD88 (C5aR1) Monoclonal Antibody-Alexa Fluor™ 488 (Thermo Fisher, Cat#53-0882-82, 20/70, RRID: AB_2811859), SiglecH Antibody-Pacific Blue™ (Biolegend, Cat#129609, 551, RRID: AB_10643868), CD172a (SIRP alpha) Monoclonal Antibody-Brilliant Violet 510™ (Biolegend, Cat#144032, P84, RRID: AB_2810411), CD197 (CCR7) Monoclonal Antibody-Brilliant Violet 605™ (Biolegend, Cat#120125, 4B12, RRID: AB_2715777), CD195 (CCR5) Monoclonal Antibody-PE (Thermo Fisher, Cat#12-1951-82, HM-CCR5 (7A4), RRID: AB_657684), CD370 (CLEC9A, DNGR1) Monoclonal Antibody-APC (Biolegend, Cat#143505, 7H11, RRID: AB_2566379), CD197 (CCR7) Monoclonal Antibody-APC (Thermo Fisher, Cat#17-1971-82, 4B12, RRID: AB_469444), CD86 Monoclonal Antibody-Brilliant Violet 421™ (Biolegend, Cat#105123, PO3, RRID: AB_2892270).

### Luminex multiplex cytokine assay

For the multiplex cytokine assay, DC suspensions (8 × 10^5^ cells per well) were cultured in six-well plates. After 24 h of incubation at 37 °C, supernatants were collected by centrifugation at 300 × g for 10min. Cytokine levels, including IL - 6, IL - 10, IL - 12p40, IL - 12p70, CCL2, CCL4, CCL5, CXCL1, tumor necrosis factor (TNF)-α, and interferon (IFN)-γ, were measured using a Luminex multiplex cytokine assay (LabEx Biotech Co.) in accordance with the manufacturer’s instructions.

### Western blotting

Cells were lysed using a radioimmunoprecipitation assay buffer containing 1% Triton X - 100, 1% sodium deoxycholate, and 0.1% sodium dodecyl sulfate (Epizyme Biotech), supplemented with 1% protease inhibitors and 1% phosphatase inhibitors (Epizyme Biotech). Protein concentrations were determined using the Omni-Easy™ bicinchoninic acid protein assay kit (Epizyme Biotech), in accordance with the manufacturer’s protocol. Equal amounts of protein from each sample were separated by sodium dodecyl sulfate–polyacrylamide gel electrophoresis and transferred onto polyvinylidene fluoride membranes (Epizyme Biotech). The membranes were blocked using a protein-free rapid blocking buffer (Epizyme Biotech). Primary antibodies were applied at a 1:1000 dilution, followed by horseradish peroxidase (HRP)-conjugated secondary antibodies at a 1:10000 dilution. Protein visualization was performed using the Omni-ECL™ chemiluminescent detection kit (Epizyme Biotech) on the ChemiDoc™ XRS+ Imaging System (Bio-Rad). Antibodies targeting the following proteins were used: IRF8 (Abcam Cat# ab85059, RRID: AB_1860896), GAPDH (Abmart Cat# M20050, RRID: AB_2936268), ZBTB46 (Proteintech Cat# 25455 - 1-AP, RRID: AB_2880088), and rabbit IgG (HRP-conjugated antibody; Proteintech Group).

### Tumor models and cell therapy

After a 3-day acclimation period, the hair on the right underarm of each mouse was shaved. For *in vivo* experiments, 8 × 10^5^ MC38 tumor cells or 5 × 10^5^ B16F10 tumor cells were subcutaneously injected into the right flank. Mice were randomized into three groups: NC group, FL/GM-DC group, and GM/IL4-DC group, with 8 mice per group. The FL/GM-DC and GM/IL4-DC groups received the first dose of DCs (2.5 × 10^6^ cells) via footpad injection on day 3, followed by two doses per week for the first 2 weeks and one dose per week for the subsequent 2 weeks. NC group mice received an equal volume of PBS at the same treatment time points. Tumor area was calculated using the formula Area = L × W, and tumor volume was calculated using the formula Volume = (L × W^2^)/2, where L represents tumor length and W represents tumor width. Tumor size was measured every 3 days using a caliper. Blinding was applied during tumor size measurements. The researchers responsible for measuring tumor volume and assessing experimental endpoints were blinded to the group allocation to ensure objectivity and reliability of the data. Mice were euthanized when tumors reached 20mm in any dimension or developed ulceration. Collected tumor samples and plotted the tumor growth curve after the experiment was completed.

After extracting the tumor, it was processed through digestion to convert it into a single-cell suspension. Subsequently, the cell surface markers were stained using antibodies against CD3, CD4 (FITC, Thermo Fisher Cat# 11 - 0041-82, GK1.5, RRID: AB_464892), CD8a (Brilliant Violet™ 421, Thermo Fisher Cat# 404 - 0081-82, 53 - 6.7, RRID: AB_2921017), and CD25 (PE-Cyanine5, Thermo Fisher Cat# 15 - 0251-82, PC61.5, RRID: AB_468733). Once staining was complete, the cells were washed and then incubated with the Foxp3 fixation/permeabilization working solution (Thermo Fisher) in the dark at 4 °C for 60 minutes. After incubation, the sample was centrifuged at 400g for 5 minutes with the addition of the permeabilization buffer (Thermo Fisher). Following this, an appropriate amount of Foxp3 antibody (PE, Thermo Fisher Cat# 12 - 5773-82, FJK - 16s, RRID: AB_465936) was added, and the sample was incubated in the dark at room temperature for 30 minutes. After the antibody incubation, the cells were washed twice with the permeabilization buffer for flow cytometry analysis.

### Immune cell depletion

To deplete F4/80^+^tumor-associated macrophages (TAMs), CD8^+^T cells, and CD4^+^T cells, mice were randomly divided into the following groups: NC group, FL/GM-DC+IgG2b group, FL/GM-DC+anti-F4/80 group, FL/GM-DC+anti-CD8a group, FL/GM-DC+anti-CD4 group, with 6 mice per group. Mice were intraperitoneally injected with 100μL of the following antibodies: anti-mouse F4/80 (Bio X Cell Cat# BE0206, CI:A3-1, RRID: AB_10949019), anti-mouse CD8a (Selleck Cat# A2102, 2.43, RRID: AB_3099521), anti-mouse CD4 (Selleck Cat# A2101, GK1.5, RRID: AB_3677296), or rat IgG2b isotype control (Selleck Cat# A2116, LTF - 2, RRID: AB_3662740). Each antibody was administered at a dose of 200 μg per mouse on the next day as DC reinfusion, with repeated injections every 3 days. The remaining treatment plan was the same as the DC anti-tumor treatment plan. Plotted the tumor growth curve after the experiment was completed.

### DC vaccination experiments

For vaccination experiments, mice were randomly divided into three groups: NC group, FL/GM-DC group, and GM/IL4-DC group, with 6 mice per group. The FL/GM-DC and GM/IL4-DC groups received two doses of DCs (2.5×10^6^ cells per injection) via footpad injection over the course of one week. Mice in the NC group were administered an equal volume of PBS. Three days after the final DC injection, mice were subcutaneously injected with either 5 × 10^5^ B16F10 cells or 8 × 10^5^ MC38 cells. Tumor sizes were measured every 3 days using a caliper. At the end of the experiment, tumor samples were collected and tumor growth curves were plotted.

### Multiplex immunohistochemistry

Freshly isolated tumors were fixed with 4% paraformaldehyde (Biosharp) at 4°C. Paraffin-embedded tumor sections were prepared in accordance with standard immunohistochemistry protocols. Sections were dewaxed after baking, and tissues were rehydrated using a graded ethanol series. Antigen retrieval was performed using an EDTA alkaline antigen retrieval solution (50×, pH 8.0, AiFang Biological) in a microwave oven. After pretreatment, tissues were blocked, and primary antibodies were applied at the following dilutions: CD4 ((Abcam Cat#ab183685, RRID: AB_2686917)), 1:3000; CD8a (Cell Signaling Technology Cat# 98941, RRID: AB_2756376)), 1:2000; CD11c (Abcam Cat# ab254183, RRID: AB_3096928), 1:3000; and Foxp3 (R and D Systems Cat#MAB8214, RRID: AB_2929004), 1:2000. Tissues were incubated with primary antibodies for 30 min at room temperature, then washed with phosphate-buffered saline plus Tween (AiFang Biological). HRP-conjugated anti-rabbit/mouse secondary antibodies (AiFang Biological) were applied and incubated for 30 min at room temperature. Target antigens were visualized using TYR - 520 and TYR - 570 chromogens. Nuclei were counterstained with 4′,6-diamidino-2-phenylindole (DAPI; AiFang Biological). Stained sections were mounted with anti-fluorescence quenching sealant (AiFang Biological), observed under a fluorescence microscope, and imaged.

### Single-cell RNA sequencing

Sample preparation: Collected *in vitro* cultured cell samples or tumor tissue samples digested with tissue digestion solution, after passing through a 40-micron filter, single cells were resuspended in Dulbecco’s phosphate-buffered saline (Biosharp) containing 2% fetal bovine serum, ensuring no doublets or aggregates and at least 90% cell viability.

Single-cell library preparation and sequencing: Using the GEXSCOPE^®^ microfluidic chip to capture single cells, Barcoding Beads with unique Cell Barcodes were added to the microwells of the chip. After cell lysis, the Barcoding Beads with Barcodes and UMIs captured mRNA by binding to the poly(A) tail on the mRNA, marking the cells and mRNA. The Barcoding Beads in the chip were collected, and the mRNA captured by the Barcoding Beads was reverse transcribed into cDNA and amplified. The cDNA was then fragmented, adapters were ligated, and other steps were taken to construct a sequencing library suitable for the Illumina sequencing platform.

Data preprocessing and integration: Raw reads were processed using CeleScope v1.5.2 to generate gene expression matrices. Quality control was performed using Seurat v3.1.2, during which cells with abnormal numbers of genes, UMIs, and high mitochondrial content were excluded, and low-expression genes were filtered out. The gene expression matrix was normalized and scaled, and 2000 variable genes were selected for PCA analysis. Harmony v1.0 was employed to remove batch effects, ensuring the data was properly integrated and prepared for further analysis.

Clustering and visualization: Cells were clustered and grouped using the Louvain clustering algorithm, grouping cells with similar gene expression profiles into a single cell cluster. The dimensionality-reduced data was visualized using UMAP.

Cell type identification and differential gene analysis: Cell-ID, a multivariate approach that extracts gene signatures for each individual cell, and hypergeometric tests(HGT) were used to identified cell types. Dimensionality reduction was performed on normalized gene expression matrix through multiple correspondence analysis, where both cells and genes were projected in the same low dimensional space. The cell type identification of each cluster was determined according to the expression of canonical markers from the reference database SynEcoSysTM (Singleron Biotechnology). DEGs were identified using Seurat’s FindMarkers function (Wilcoxon test, default settings), selecting genes expressed in at least 10% of cells and with logFC > 0.25. Adjusted p value was calculated by Bonferroni Correction and the value 0.05 was used as the criterion to evaluate the statistical significance.

Enrichment analysis: By conducting enrichment analysis on differentially expressed genes among various cell types, we used the clusterProfiler software to perform Gene Ontology (GO) pathway enrichment analysis on the gene sets. The P-value was calculated by hypergeometric distribution method (the criterion for significant enrichment is P-value < 0.05).

Gene set scoring: We used the UCell v1.1.0 for gene set scoring, based on the Mann-Whitney U statistic to rank gene expression levels.

Pseudotime trajectory analysis: We employed Monocle3 to determine the temporal changes in the expression levels of specific genes.

TCR clonotype analysis: We used Cell Ranger (v4.0.0) vdj pipeline for TCR clonotype assignment.

### Statistical analysis

Statistical analysis was performed using GraphPad Prism 8 software. Data are presented as mean ± standard error of the mean (SEM). Statistical significance was assessed using unpaired Student’s t-tests, one-way analysis of variance (ANOVA), or two-way ANOVA with multiple comparisons, as specified in the figure legends. Statistical significance was indicated as “ *p<0.05, **p<0.01, ***p<0.001, and ****p<0.0001”.

## Results

### Phenotyping distinct subpopulations in FLT3L/GM-CSF based culture method with protein expression

To phenotype distinct subpopulations in the FLT3L/GM-CSF-based culture method with protein expression, we performed a detailed flow cytometry-based analysis comparing FL/GM-DC and GM/IL4-DC cultures.

First, we performed initial gating on the cell population based on forward scatter (FSC) and side scatter (SSC), parameters that reflect the physical characteristics of cells. This allowed us to identify two distinct subpopulations morphologically: one characterized as low forward scatter and high side scatter (FSC^low^SSC^high^), and another as high forward scatter and low side scatter (FSC^high^SSC^low^). We initially hypothesized that the FSC^low^SSC^high^ population might represent smaller cells with relatively simple internal structures, such as progenitor cells or early differentiating cells, while the FSC^high^SSC^low^ population might represent larger cells with more complex internal structures, such as differentiated DCs.

To verify this preliminary hypothesis based on physical parameters, we applied classic DC differentiation markers—the cell surface proteins CD11c and MHCII. The analysis results showed that, after excluding background cells not expressing these two markers (MHCII^−^CD11c^−^), 55.03% of the cells in the FSC^low^SSC^high^ population exhibited intermediate levels of MHCII expression but lacked CD11c. This suggests that this population might be in the early stages of DC differentiation, where MHCII function has begun to be initiated, but the typical DC lineage marker CD11c has not yet been fully expressed. To further clarify the identity of these FSC^low^SSC^high^, MHCII^+^CD11c^−^ cells, we examined them for cDC lineage markers, including XCR1 (used to identify the cDC1 subset) and CLEC4a4 (used to identify the cDC2 subset) (33, 34). The results revealed that 15.3% of these MHCII^+^ cells co-expressed XCR1 and CLEC4a4. This finding is intriguing; it suggests that these cells might represent a precursor subset or a transitional state within the DC lineage: they have already begun expressing cDC lineage-related markers, but these markers may be partially or co-expressed before CD11c is fully upregulated to reach the level of mature DCs. In contrast, within the FSC^high^SSC^low^ cell population, a significant proportion (32.7%) consisted of cells clearly expressing CD11c and highly expressing MHCII (CD11c^+^MHCII^high^). By further gating using XCR1 and CD172a (another marker commonly used to distinguish cDC subsets), we could subdivide these cells into distinct subsets whose phenotypes were highly consistent with known, fully differentiated cDC1 and cDC2 subsets (**Supplementary Figure 1a**). This strongly supports our initial hypothesis that the FSC^high^SSC^low^ population represents differentiated DCs. Based on these analyses, in subsequent in-depth analyses, we will focus primarily on the FSC^high^SSC^low^ cell population, which exhibits a more mature and differentiated phenotype, to investigate in detail the characteristics and functions of differentiated DCs.

As gating solely on CD11c^+^MHCII^+^cells failed to efficiently exclude monocytic cells with partial DC-like characteristics, we designed a novel gating strategy, monocyte-lineage cells expressing CD88 were excluded, and pDCs were gated by SiglecH expression. Subsequently, immature and mature conventional DC (cDC) subsets were differentiated by assessing surface CCR5 and CCR7 expression levels (mature DC express higher CCR7 for migration to lymph nodes, whereas immature DC predominantly express CCR5 facilitating peripheral localization.

Previous studies reported that low-dose GM-CSF could synergize with FLT3L to enhance cDC induction by promoting DC progenitor differentiation and proliferation, partly through inhibition of Stat5-mediated Irf8 expression, thereby suppressing pDC differentiation (35). Consistent with this, the addition of 10 ng/mL GM-CSF to FLT3L-based cultures increased the proportion of differentiated DCs (from 19.9% to 38.4%) without significantly altering FSC/SSC properties, promoted expansion of the XCR1^+^cDC1 subset, and correspondingly decreased the proportion of CD172a^+^cDC2 subset (**Supplementary Figure 1b**). In contrast, high concentrations of GM-CSF (100 ng/mL) induced a pronounced shift toward monocyte/macrophage-like differentiation, indicated by increased FSC/SSC values, elevated CD88 expression, loss of cDC1 marker expression, and a marked expansion of CD172a^+^cells (data not shown).

**Figure 1:**
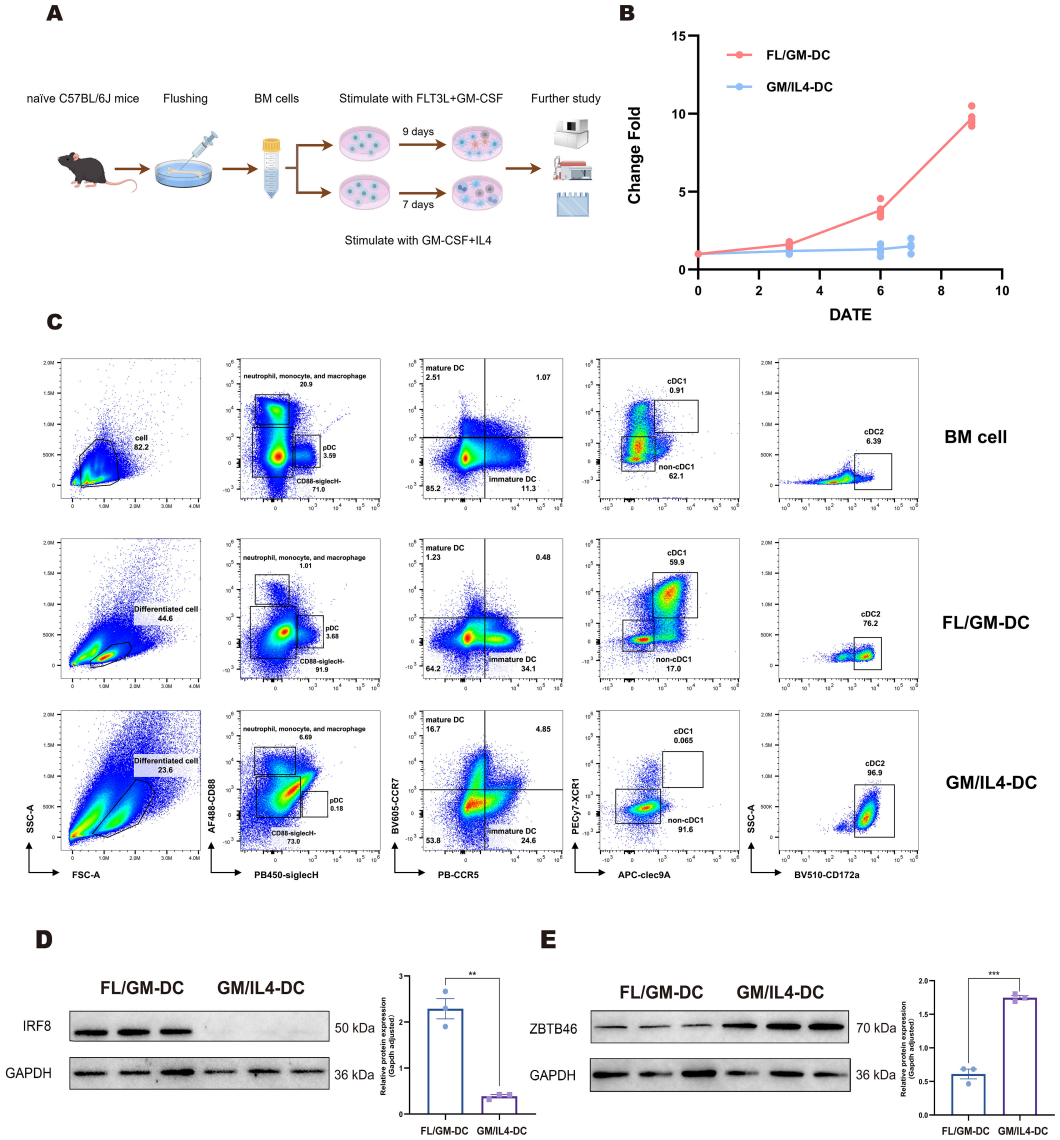
Cells induced by the FLT3L-based culture method partially expressed cDC1 phenotype. **(a)** Schematic of the procedure to generate FL/GM-DCs and GM/IL4-DCs from mouse bone marrow cells. **(b)** FL/GM-DC (red) and GM/IL4-DC (blue) amplification change fold during the culture process. The vertical axis represents the change fold, and the horizontal axis represents the number of days. **(c)** Flow cytometry for comparing the distribution and characteristics of different cell subpopulations in mouse BM cells, FL/GM-DCs, and GM/IL4-DCs. **(d, e)** Western blotting analysis showed the protein expression levels of transcription factors IRF8 and ZBTB46.

Therefore, based on these observations, we adopted the combination of FLT3L supplemented with 10 ng/mL GM-CSF for subsequent DC culture experiments. Alternatively, in the GM-CSF/IL-4 based method, BM cells were cultured for 7 days in the presence of high concentrations of GM-CSF and IL - 4. (**Figure 1a**). Upon completion of the culture period, FL/GM-DCs exhibited a nine-fold expansion, whereas GM/IL4-DCs displayed a substantially lower yield (1.45-fold expansion) (**Figure 1b**). This discrepancy likely resulted from the greater proportion of adherent cells generated in the GM/IL4-DC culture and the inflammatory environment mimicked by the GM-CSF and IL - 4 method. Sustained stimulation in this setting may induce apoptosis, leading to lower cell yields.

Then we compared the phenotypes of DC between GM/IL4-DC and FL/GM-DC with our new panel. We found that GM/IL4-DC closely resembled FLT3L culture supplemented with 100 ng/mL GM-CSF, further demonstrating that GM-CSF dominates the differentiation pathway at higher concentrations. The proportion of differentiated DC in GM/IL4-DC was 23.6%, significantly higher SSC than FL/GM-DC, among which 6.69% were CD88^+^, and almost no SiglecH^+^pDC subset was detected. Within the CD88^-^SiglecH^-^cells, virtually all cells expressed CD172a, and cDC1 markers were completely absent (**Figure 1c**). These results indicated distinct DC subset phenotypes between FL/GM-DC and GM/IL4-DC cultures.

Western blotting analysis demonstrated distinct expression patterns of the transcription factors Irf8 and Zbtb46, which are critical regulators of cDC differentiation. FL/GM-DC exhibited substantially higher Irf8 expression relative to GM/IL4-DC, whereas GM/IL4-DC displayed higher Zbtb46 expression, likely reflecting a predominance of cDC2 or mixed DC/macrophage populations rather than a specific cDC1 signature (**Figures 1d, e**).

These findings suggest that although both induction methods can generate cDC populations, FL/GM-DCs preferentially differentiate into the cDC1 lineage. In contrast, GM/IL4-DC induction predominantly produces cDC2/Macrophage cells.

### Single-cell sequencing reveals heterogeneous DC clusters in FL/GM-DC

**Figure 2:**
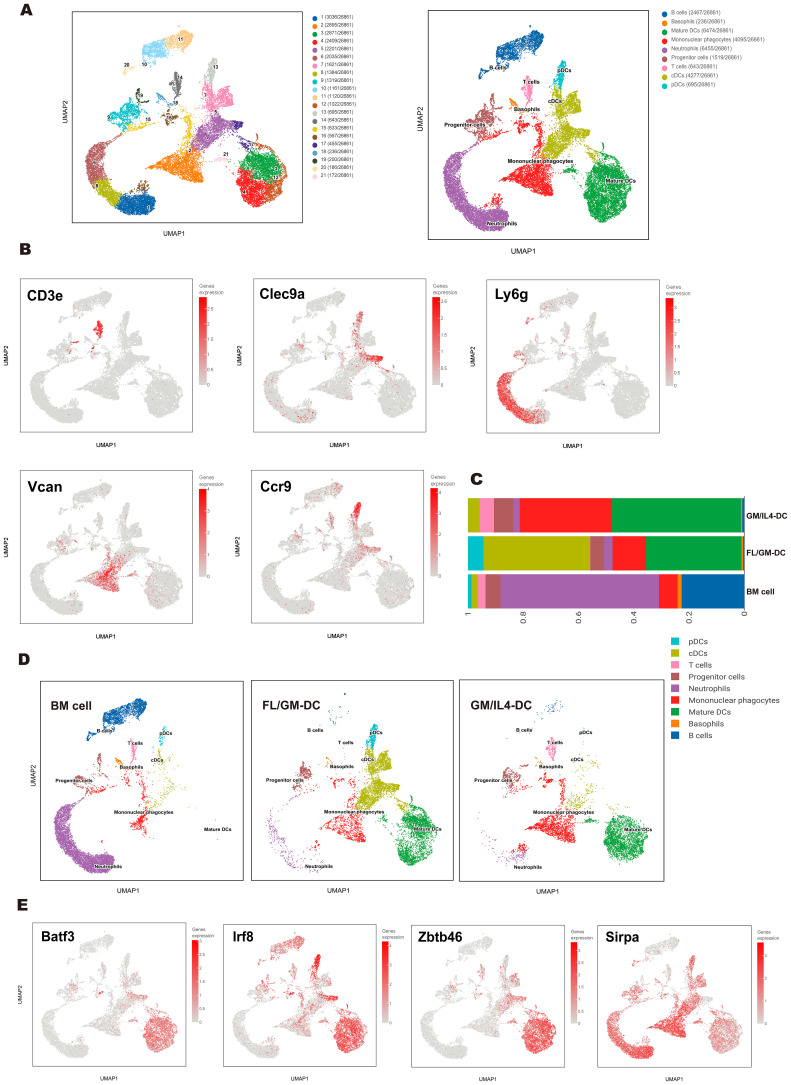
Single-cell transcriptomics revealed a heterogeneous cell population of FL/GM-DCs. **(a)** UMAP plot depicted various subpopulations in distinct colors (left panel). UMAP visualization of clusters based on characteristic gene expression annotations (right panel). **(b)** Expression of marker genes of each subgroup on the UMAP. **(c)** Stacked bar chart showed the proportions of each cluster across the BM cell group, FL/GM-DC group, and GM/IL4-DC group. **(d)** UMAP distribution plots showed the proportions of each cluster in the BM cell group, FL/GM-DC group and GM/IL4-DC group. **(e)** Expression of cDC-related transcription factors and markers on UMAP plot.

To further characterize the differentiation trajectories and cellular heterogeneity within DC populations. We analyzed a total of 10,303 cells from FL/GM-DC cultures, 9,820 cells from GM/IL4-DC cultures, and 10,922 cells from freshly isolated bone marrow (BM). Uniform manifold approximation and projection (UMAP) analysis of pooled samples identified 21 distinct cell clusters, annotated into nine major subsets based on canonical marker genes and differential gene expression patterns (**Figure 2a**). These subsets included progenitor cells (clusters 9 and 19; expressing *Cdc20* and *Ccnb2*), T cells (cluster 14; expressing *Cd3e*, *Trbc2*, *Trac*, and *Il7r*), B cells (clusters 10, 11, and 20; expressing *Cd19*, *Ms4a1*, and *Cd79b*), mononuclear phagocytes (clusters 2, 15, and 16; expressing *Vcan*, *Msr1*, *Cd14*, and *Mrc1*), neutrophils (clusters 1, 6, and 8; expressing *Csf3r*, *Lcn2*, and *Ly6g*), plasmacytoid dendritic cells (pDCs; cluster 13; expressing *Siglech*, *Ccr9*, and *Tcf4*), conventional dendritic cells (cDCs; clusters 5, 7, and 17; expressing *Clec4a4*, *Clec9a*, *Xcr1*, and *Zbtb46*), and mature DCs (clusters 3, 4, 12, and 21; expressing *Fscn1*, *Ccr7*, and *Ccl5*) (**Figure 2b**). The accuracy of these annotations was further validated by analyzing differentially expressed genes (DEGs) among the identified subsets (**Supplementary Figure 2a**).

Comparison of FL/GM-DC and GM/IL4-DC populations revealed significant differences in cell composition, particularly in DC subtypes and activation states. FL/GM-DCs were predominantly composed of cDCs (38.59%), pDCs (5.6%), and mature DCs (34.6%), with mononuclear phagocytes comprising 12.1% of the total population. In contrast, GM/IL4-DCs consisted primarily of mature DCs (46.7%) and mononuclear phagocytes (33.3%), while cDCs represented only 4.2% of the population, and pDCs were nearly absent (**Figures 2c, d**). Interestingly, approximately 5.1% of the GM/IL4-DC culture population consisted of T cells, whereas no T cells were detected in FL/GM-DC cultures. To validate this observation, we performed flow cytometry analysis, which confirmed the presence of CD3^+^ T cells in GM/IL4-DCs, while none were detected in FL/GM-DCs. Furthermore, adjusting the concentration of GM-CSF (0 to 100 ng/mL) in the FLT3L culture system did not alter this outcome. These findings suggest that IL - 4 may promote the emergence of T cells in GM/IL4-DC cultures, possibly by influencing the differentiation of precursor cells (**Supplementary Figure 2b**). However, this conclusion should be interpreted with caution, as other factors—such as contamination during cell culture—cannot be ruled out. Therefore, we propose that IL - 4 may play a role in this process, but this remains a hypothesis requiring further functional validation.

Mature DCs constituted a significant proportion of both FL/GM-DC and GM/IL4-DC populations. In mature DCs, transcription factors critical for cDC differentiation (*Zbtb46*, *Irf8*, and *Batf3*) remained highly expressed; however, the expression levels of canonical cDC1 and cDC2 markers (*Xcr1*, *Clec9a*, *Cd209a*, and *Sirpa*) were significantly downregulated, making it challenging to determine the precise lineage origin of mature DCs (**Figure 2e**). To investigate how different culture methods influence the functional state of mature DCs, we compared differentially expressed genes (DEGs) between FL/GM-DC- and GM/IL4-DC-derived mature DC subsets (**Supplementary Figure 2c**). Among the top upregulated genes in FL/GM-DC-derived mature DCs, *Il12b* and *Cd40* were highly expressed, indicating a more immunostimulatory phenotype that facilitates DC-T cell interactions. *Isg15* expression suggested that type I interferon signaling played a crucial role in FL/GM-DC activation. Additionally, *Apol7c*, a cDC1-specific apolipoprotein involved in antigen cross-presentation, was also found to be upregulated in a subset of mature DCs, although its precise functional implications remain unclear (36). Conversely, mature DCs from GM/IL4-DC cultures exhibited upregulation of *Fabp5* and *Vdr*, both of which promote fatty acid oxidation and contribute to an immunotolerant phenotype. High *Slamf1* expression further reinforced this tolerogenic state, while elevated *Ccl17* expression suggested an enhanced ability to recruit CCR4^+^ regulatory T cells (Tregs). Gene Ontology (GO) and Wikipathway enrichment analyses further indicated that mature DCs from FL/GM-DCs were more proficient in promoting T-cell activation and expansion compared to their GM/IL4-DC counterparts (**Supplementary Figures 2d, e**). To further quantify these functional differences, we applied UCell-based gene set scoring to evaluate distinct gene signatures in FL/GM-DC- and GM/IL4-DC-derived mature DCs. The gene sets included: Maturation signature (*Cd40*, *Cd80*, *Cd86*, *Ccr7*), Antigen presentation signature (*Cd74*, *H2-Eb1*, *H2-Aa*, *H2-Ab1*), Pro-inflammatory signature (*Ccl20*, *Il1b*, *Ccl4*, *Ccl3*, *Cxcl1*, *Areg*, *Cxcl2*, *Cxcl3*, *Hbegf*, *Ereg*, *Cxcl5*), Lipid metabolism signature (*Ctsb*, *Ctsd*, *Alox5ap*, *Fabp4*, *Fabp5*, *Acp5*, *Lpl*, *Trem2*, *Ctsl*), Interferon response signature (*Cxcl11*, *Isg20*, *Cxcl10*, *Casp4*, *Casp1*, *Ifitm1*, *Ifitm3*, *Cxcl9*, *Isg15*, *Ifit1*, *Ifit2*, *Ifi44l*, *Ifit3*). Scoring results revealed that mature DCs in FL/GM-DC cultures exhibited superior scores in Antigen Presentation and Interferon Response signatures, whereas mature DCs from GM/IL4-DC cultures scored higher in Maturation and Lipid Metabolism signatures (**Supplementary Figures 3a, b**).

**Figure 3:**
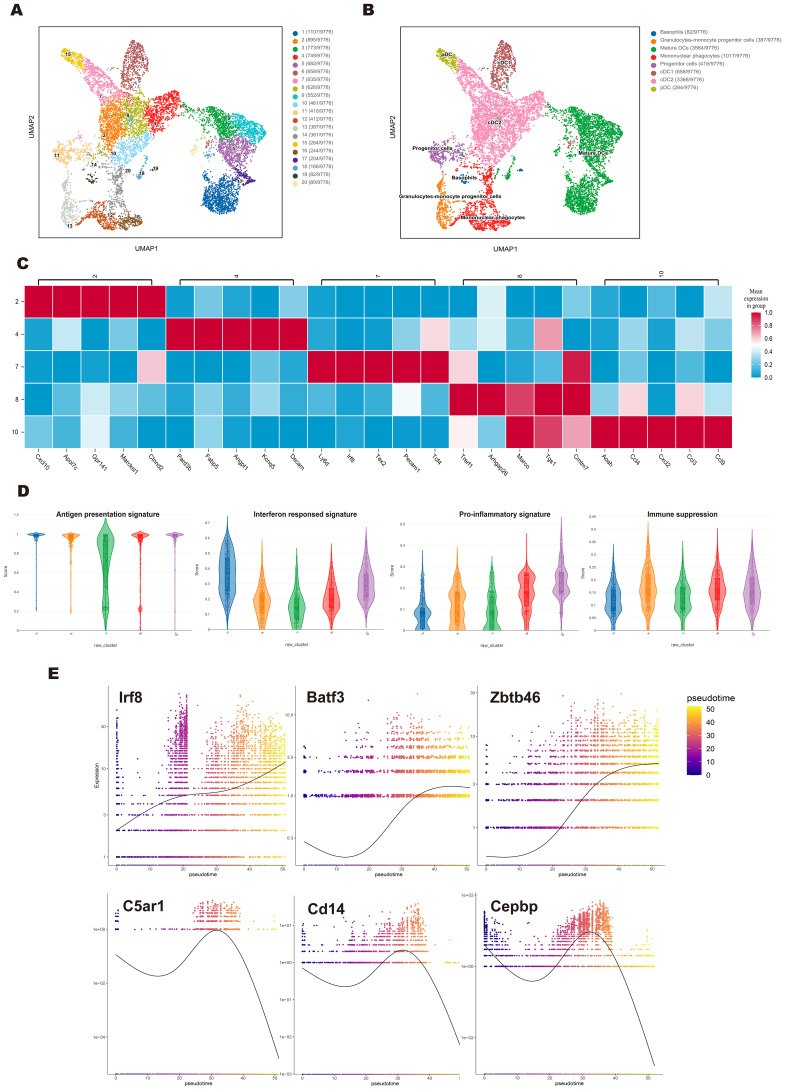
Single-cell sequencing revealed the cDC subpopulation and developmental trajectory in the FL/GM-DC group. **(a)** Based on the FL/GM-DC group, UMAP plot depicted various subpopulations in distinct colors. **(b)** Based on the FL/GM-DC group, UMAP visualization of clusters based on characteristic gene expression annotations. **(c)** Heatmap represented the expression of the top 5 genes in different clusters of cDC2 in the FL/GM-DC group. **(d)** Violin plot displayed the Ucell-based gene set scores to evaluate the characteristics of different gene signatures in different clusters of cDC2 in FL/GM-DC group. **(e)** The temporal expression trends of key cDC1 genes *Irf8*, *Batf3*, *Zbtb46*, and monocyte key genes *C5ar1*, *Cd14*, *Cebpb* in the FL/GM-DC group. The x-axis indicated the chronological order of cells, while the y-axis represented the expression levels of these key genes.

To further investigate subpopulation and developmental trajectories of cDC, we performed a separate analysis of FL/GM-DC data. UMAP analysis identified 20 distinct cell clusters, which were initially annotated into 8 subsets based on characteristic gene expression profiles (**Figures 3a, b**). The major subsets included pDCs (3%), cDCs (41.1%), and mature DCs (36.5%), displaying a slightly different composition compared to the pooled analysis (**Supplementary Figure 3c**). As expected, cDC1 (6.7%) was readily identifiable based on the canonical markers *Xcr1*, *Clec9a*, and *Cxcl9*. In contrast, cDC2 (34.4%) exhibited significant heterogeneity, segregating into five distinct clusters.

To further delineate the functional characteristics of cDC2 subsets, we conducted DEG analysis across all cDC2 clusters (**Figure 3c**). Cluster 2 exhibited high expression of type I interferon (IFN)-stimulated genes, including *Isg15*, *Ifi205*, and *Ifi30*. Additionally, *Irf5*, *Irf7*, *Ly6a*, and *Cxcl10*, which are also associated with IFN responses, were significantly upregulated in this cluster. These transcriptional features partially overlap with those of ISG^+^ DCs, which have been suggested to play a role in CD8^+^ T cell activation (37). While this raises the possibility that cluster 2 may exhibit similar immunostimulatory properties, further functional studies are required to confirm such a role. Interestingly, *Apol7c*, a lipid-associated gene typically considered a hallmark of cDC1, was also highly expressed in Cluster 2. Given that *Apol7c* has been reported to promote cross-presentation through phagosomal rupture, its expression further suggests that this cluster may play a key role in enhancing CD8^+^ T cell responses. Cluster 7, positioned between pDCs and cDC subsets, expressed both pDC- and cDC-associated genes, including *Ly6d*, *Tcf4*, *Irf8*, and *Zbtb46*, with characteristic upregulation of *Cx3cr1*, *Lgals3*, and *Ms4a6c*. These features are reminiscent of transitional DCs (tDCs) or pDC-like cells, which are believed to differentiate from pro-pDCs (38). Cluster 10, closely aligned with mononuclear phagocytes, exhibited a phenotype resembling inflammation-induced macrophages, with high expression of multiple pro-inflammatory cytokines, including *Cxcl2*, *Ccl9*, *Il12b*, *Tnf*, and *Ccl3-5*. Cluster 4, which was adjacent to mature DCs, exhibited elevated expression of Fabp5, Fscn1, and Pard3b, genes linked to cell migration. Notably, St8sia6, a marker associated with immunosuppressive functions, was selectively upregulated in this cluster. These findings raise the possibility that cluster 4 may possess a tolerogenic phenotype, although further functional validation is required to confirm this hypothesis. Cluster 8 displayed strong expression of *Marco*, *Cmtm7*, *Pou2f2*, and *Btla*, supporting its role in promoting DC-mediated immune tolerance.

To further assess the functional characteristics of cDC2 subsets, we performed UCell-based gene set scoring across different clusters. Except for Cluster 7, all cDC2 clusters exhibited high scores in Maturation and Antigen Presentation signatures. Notably, Cluster 2 had the highest score in the Interferon Response signature, Cluster 10 scored highest in the Pro-inflammatory signature, Cluster 4 exhibited a slight increase in the Immune Suppression signature (*Cd274*, *Btla*, *St8sia6*, *Ido1*, *Il10*, *Socs1*, *Socs3*, *Nr4a3*, *Nr4a2*, *Nr4a1*, *Lgals1*) (**Figure 3d**). Collectively, these findings highlight the substantial heterogeneity within cDC2 subsets in FL/GM-DC cultures.

Our data suggest the existence of two distinct maturation trajectories in cDCs. In addition to the conventional maturation process—characterized by downregulation of antigen presentation-related genes, upregulation of co-stimulatory molecules (*Cd80*, *Cd40*, and *Cd86*), and CCR7-mediated lymph node migration—we observed an alternative maturation pattern in IFN-treated, *in vitro*-differentiated cDCs (39). In this pattern, *Cd86* (but not *Cd80* or *Cd40*) and *Ccr7* were highly induced, while antigen presentation-related genes remained highly expressed, accompanied by upregulation of the T-cell chemoattractants *Cxcl9* and *Cxcl10*. These gene expression changes imply a potentially distinct immune-stimulatory role for this subset. Furthermore, high *Apol7c* expression was noted in these cells, which may enhance cross-presentation of antigens via phagosomal rupture, although this requires functional validation. Notably, this IFN-associated maturation pattern was observed in both cDC1 and cDC2 subsets, suggesting it may be a shared feature within the cDC lineage (**Supplementary Figure 3d**).

Finally, we performed pseudotime trajectory analysis to assess the dynamic expression changes of genes associated with cDC differentiation over time. Notably, FL/GM-DC pseudotime analysis revealed a sustained upregulation of cDC1-related genes, including *Irf8*, *Batf3*, and *Zbtb46*, whereas monocyte-associated markers (*C5ar1*, *Cd14*, and *Cepbp*) progressively decreased. These findings further support the advantage of the FLT3L pathway in promoting cDC1 differentiation (**Figure 3e**).

### Ex vivo-differentiated FL/GM-DCs effectively activate anti-tumor immunity *in vitro*

DCs serve as a critical link between innate and adaptive immunity, enhancing anti-tumor responses by processing and presenting tumor antigens, expressing co-stimulatory molecules, secreting chemokines for tumor homing and lymph node migration, and producing interleukins. We evaluated these characteristics in FL/GM-DCs and GM/IL4-DCs through *in vitro* experiments.

**Figure 4:**
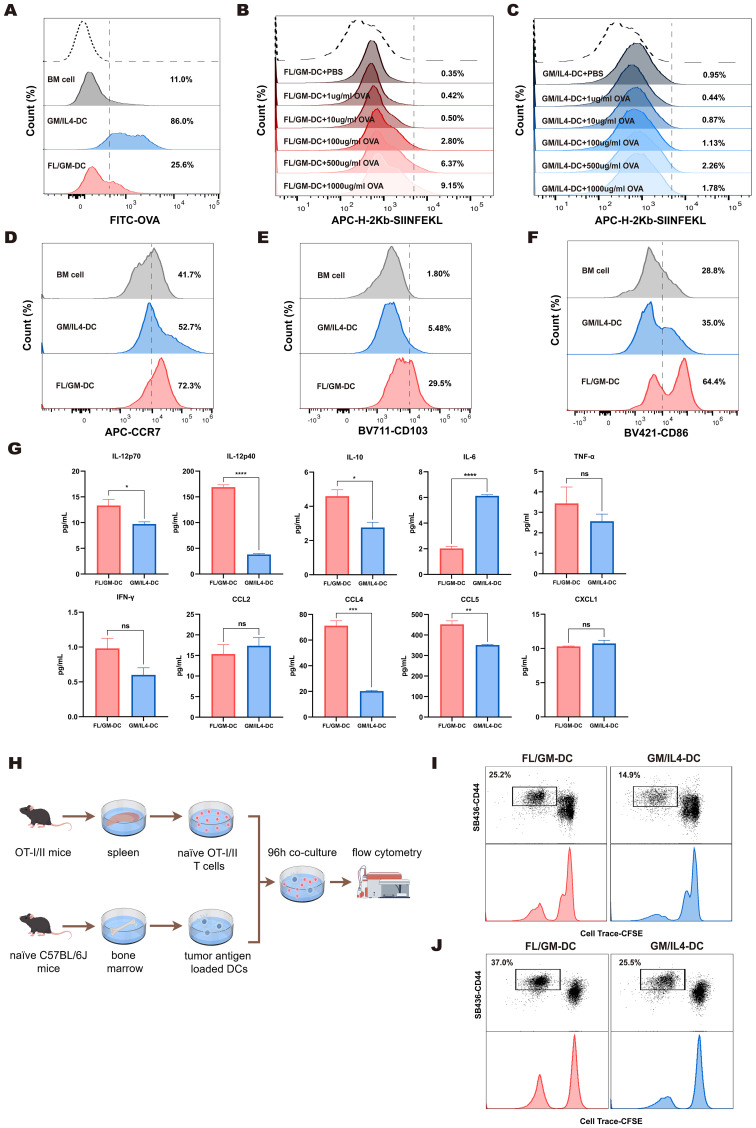
FL/GM-DCs enhance the anti-tumor immune response. **(a)** BM cells, FL/GM-DCs, and GM-DCs were cultured in equal numbers, and OVA-FITC was added for 4 h at 37 °C. OVA uptake was quantified using flow cytometry by first gating on cells based on MHCII and CD11c expression, and the results are displayed as a histogram. Each group contained at least n = 3 biological replicates. **(b, c)** FL/GM-DCs **(b)** and GM/IL4-DCs **(c)** were cultured in equal numbers and co-cultured with OVA at concentrations of 1μg/ml, 10μg/ml, 100μg/ml, 500μg/ml, and 1000μg/ml at 37 °C for 24 hours. The expression levels of H - 2Kb OVA257 – 264 on DCs were measured by flow cytometry. Each group contained at least n = 3 biological replicates. **(d)** Flow cytometric analysis of expression levels of the chemokine receptor CCR7, gated within MHCII+CD11c+ cells. The analyzed cells were stimulated with polyI:C and R484 prior to analysis. Each group contained at least n = 3 biological replicates. **(e)** Flow cytometric analysis of expression levels of the Integrin CD103, gated within MHCII^+^CD11c^+^ cells. Each group contained at least n = 3 biological replicates. **(f)** Flow cytometric analysis of expression levels of the costimulatory molecule CD86, gated within MHCII^+^CD11c^+^ cells. Each group contained at least n = 3 biological replicates. **(g)** Cytokine and chemokine levels, including IL - 12p70, IL - 12p40, IL - 10, IL - 6, TNF-α, IFN-γ, CCL2, CCL4, CCL5, and CXCL1, were measured in the supernatants of FL/GM-DC and GM/IL4-DC cultures after antigen stimulation and maturation for 72 h (n=3). **(h)** Schematic of T cell proliferation assays using CFSE-labeled OT-I and OT-II T cells co-cultured with B16-OVA tumor lysate-pulsed DCs. **(i)** Flow cytometric analysis of CD8^+^ T cell proliferation and activation after 96 h of co-culture. **(j)** Flow cytometric analysis of CD4^+^ T cell proliferation and activation after 96 h of co-culture.

To assess the ability of DCs to internalize exogenous antigens, we co-cultured FITC-labeled OVA protein with FL/GM-DCs and GM/IL4-DCs. The results indicated that both FL/GM-DCs and GM/IL4-DCs exhibit superior antigen uptake capabilities compared to BM cells. However, when assessing phagocytic activity between the two, we observed that FL/GM-DCs have a weaker phagocytic capacity than GM/IL4-DCs (**Figure 4a**, **Supplementary Figure 4a**). GM/IL4-DCs, generated with GM-CSF and IL - 4, may exhibit strong phagocytic capacity, particularly for protein antigens such as OVA, likely through the upregulation of phagocytosis-related receptors including Fcγ receptors and complement receptors (40). The underlying mechanism may involve GM-CSF-induced activation of the STAT5 and PI3K/Akt pathways, along with IL - 4-driven STAT6 signaling, which together promote cytoskeletal rearrangement and phagosome formation (41–43). In contrast, FL/GM-DCs, differentiated with FLT3L combined with low-dose GM-CSF, are more prone to developing into the cDC1 subset. These cells may express lower levels of phagocytic receptors such as DEC - 205 and instead prioritize cross-presentation via MHC class I molecules (44). Additionally, FLT3L primarily promotes DC maturation and survival through the STAT3 and MAPK pathways, rather than enhancing phagocytic function (45).

In the subsequent study, we conducted a detailed evaluation of the cross-presentation function of FL/GM-DCs and GM/IL4-DCs under stimulation with varying concentrations of OVA. The experimental data indicated that with increasing concentrations of OVA, there is a significant upward trend in the expression level of H - 2Kb-SIINFEKL in FL/GM-DCs (**Figure 4b**, **Supplementary Figure 4b**). In contrast, GM/IL4-DCs generally exhibited lower levels of H - 2Kb-SIINFEKL expression under the same conditions, and no significant differences in expression were observed between different concentrations of OVA treatment groups (**Figure 4c**, **Supplementary Figure 4c**).

After acquiring tumor antigens, mature DCs migrate to lymph nodes by expressing the CCR7 chemokine receptor, which enables efficient antigen presentation to T cells via MHC complexes. Migratory cDC1 cells are specifically characterized by CD103 expression. Using flow cytometry, we assessed CCR7 and CD103 protein levels in CD11c^+^MHCII^+^cells from both FL/GM-DCs and GM/IL4-DCs. We found that FL/GM-DCs exhibited more significant levels of CCR7 (72.3% vs. 52.7%) and CD103 proteins (29.5% vs. 5.48%) (**Figures 4d, e**, **Supplementary Figures 4d, e**).

Effective T cell activation requires antigen presentation in combination with co-stimulatory signals and cytokines. Our scRNA sequencing data demonstrated high expression of co-stimulatory signal genes such as *Cd86*, *Cd80*, *Icosl*, and *Tnfsf4* in both FL/GM-DCs and GM/IL4-DCs. Flow cytometry revealed elevated surface CD86 protein levels in CD11c^+^MHCII^+^cells from both DC types. It was worth noting that FL/GM-DCs exhibit a more significant increase in this metric (64.4% vs. 35.0%) (**Figure 4f**, **Supplementary Figure 4f**). Additionally, cytokine/chemokine multiplex analysis showed that FL/GM-DCs enhance anti-tumor immune responses by releasing cytokines such as IL - 12 and IFN-γ while producing lower levels of IL - 6 relative to GM/IL4-DCs (**Figure 4g**).

To validate the ability of ex vivo-differentiated DCs to present tumor antigens and activate T cells, we pulsed FL/GM-DCs and GM/IL4-DCs with B16-OVA tumor lysates, then co-cultured those pulsed DCs with CD8^+^T cells isolated from OT-I transgenic mice (**Figure 4h**). After 96 h, FL/GM-DCs demonstrated superior antigen-specific CD8^+^T cell activation, indicated by higher CD44 expression levels. Furthermore, FL/GM-DCs induced a significantly greater proportion of proliferating T cells relative to GM/IL4-DCs (25.2% vs. 14.9%) (**Figure 4i**).

In a parallel experiment involving OT-II transgenic mice, we observed similar results. When FL/GM-DCs loaded with B16-OVA tumor lysates were co-cultured with CD4^+^T cells that had been isolated from OT-II mice (**Figure 4h**), we detected a significant increase in the activation and proliferation of CD44^+^CD4^+^T cells relative to those co-cultured with GM/IL4-DCs (37% vs. 25.5%) (**Figure 4j**). This result confirms that, compared to GM/IL4-DCs, FL/GM-DCs have a preferential ability to promote the activation and expansion of both CD8^+^T cells and CD4^+^T cells.

These findings indicate that both FL/GM-DCs and GM/IL4-DCs effectively present antigens and activate T cells, which are critical for initiating adaptive immune responses. However, FL/GM-DCs exhibit a stronger immunostimulatory effect compared with GM/IL4-DCs.

### Enhanced tumor infiltration and lymph node homing of FL/GM-DCs with prolonged survival *in vivo*

Compared with T lymphocytes, myeloid immune cells such as DCs demonstrate greater plasticity and are more influenced by the tumor microenvironment, often resulting in functional impairment that can be observed *in vitro*. Additionally, current evidence suggests that fully differentiated DCs, particularly when activated, have a shorter lifespan. These factors require consideration during the development of DC-based therapies.

**Figure 5:**
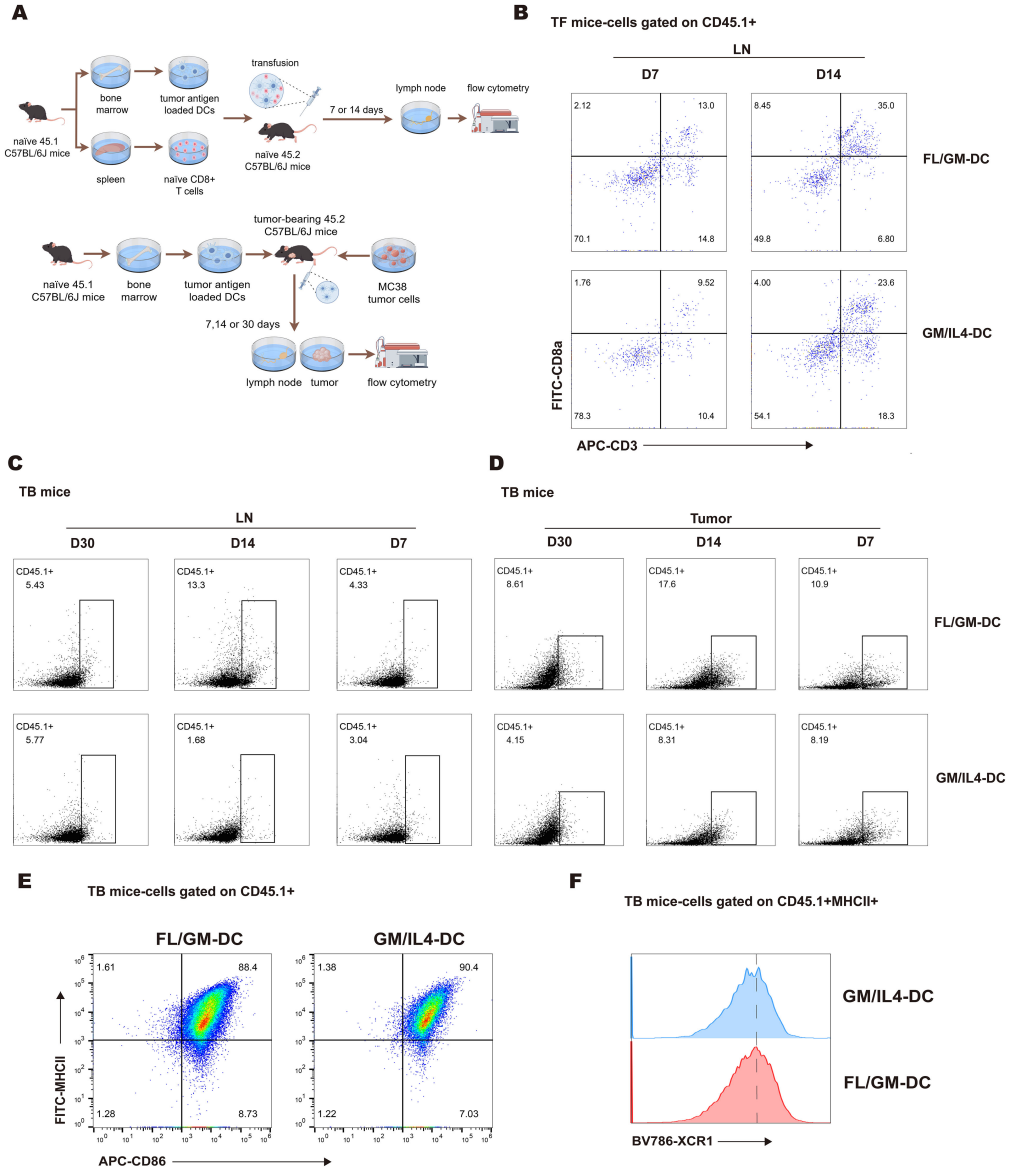
FL/GM-DCs exhibit migration and long-term survival *in vivo*. **(a)** Experimental protocol for tracking the fate of adoptively transferred FL/GM-DCs and GM/IL4-DCs in tumor-free mice (above) and MC38 tumor-bearing mice (below). In this experiment, each group consisted of 3 mice. **(b)** Flow cytometric analysis of CD45.1^+^ CD8^+^ T cell infiltration in the lymph nodes of tumor-free mice at 7 or 14 days after adoptive transfer of DCs and T cells. **(c)** Flow cytometric analysis of the proportion of CD45.1^+^ cells in the lymph nodes of MC38 tumor-bearing mice on days 7, 14, and 30 post-transfer. **(d)** Flow cytometric analysis of the proportion of CD45.1^+^ cells in MC38 tumor tissue on days 7, 14, and 30 post-transfer. **(e)** Flow cytometric analysis of infiltrating CD45.1^+^ DCs (MHCII^+^CD86^+^) in MC38 tumor tissue after adoptive transfer of CD45.1^+^ DCs. **(f)** Flow cytometry histogram showing XCR1 expression on MHCII^+^CD86^+^ DCs.

We investigated whether ex vivo-derived DCs could efficiently migrate to lymph nodes or tumor tissues and survive *in vivo*. FL/GM-DCs and GM/IL4-DCs were generated from bone marrow cells of CD45.1 transgenic mice. Subsequently, CD45.1^+^DCs and T cells (isolated from the spleens of CD45.1 mice) were adoptively transferred into CD45.2 recipient mice via footpad injection. Lymph nodes were harvested 7 and 14 days after transfer, then analyzed using flow cytometry (**Figure 5a** above). Over time, we observed a pronounced increase in the number of CD45.1^+^CD8^+^T cells in the lymph nodes of mice in the FL/GM-DC group relative to the GM/IL4-DC group. This finding indicates that ex vivo-derived FL/GM-DCs exhibited superior lymph node migration and more efficient CD8^+^T cell activation compared with GM/IL4-DCs (**Figure 5b**).

Next, we evaluated the tumor infiltration and lymph node homing capacity of induced DCs in tumor-bearing mice over time (**Figure 5a** below). The analysis revealed a significantly higher number of tumor-infiltrating CD45.1^+^cells in the FL/GM-DC group compared to the GM/IL4-DC group at all three time points (**Figure 5d**). Nearly all infiltrated CD45.1^+^cells exhibited high expression levels of MHCII and CD86 (**Figure 5e**), with comparable proportions of XCR1^+^cells in both groups (**Figure 5f**). These results suggest that FL/GM-DCs have a superior ability to accumulate and survive within the tumor microenvironment. The peak number of CD45.1^+^cells in the tumor was observed on day 14; it had decreased by ~50% by day 30. A similar trend was noted in the lymph nodes of the FL/GM-DC group, where the number of infiltrated CD45.1^+^cells peaked at day 14 and was significantly higher than the number in the GM/IL4-DC group. By day 30 post-adoption, the number of CD45.1^+^cells in the lymph nodes declined by more than 50%, reaching levels comparable to those of the GM/IL4-DC group (**Figure 5c**). These findings suggest that in a tumor-bearing mouse model, FL/GM-DCs show enhanced tumor infiltration and lymph node homing capacities relative to GM/IL4-DCs.

### FL/GM-DCs demonstrate superior anti-tumor effects across different tumor models

Multiple studies had shown that immune cell interactions are important in establishing the systemic anti-tumor immune response needed to achieve immunotherapy success (46, 47). The immune composition of the tumor microenvironment significantly varies among tumor types, with differences in the extent of immune infiltration and diversity. The B16F10 and MC38 tumor models are widely used but immunologically distinct. B16F10 tumors are characterized by a low mutation burden, whereas MC38 tumors exhibit a high mutation burden. In terms of immune cell composition within the tumor microenvironment, the MC38 model is heavily infiltrated by immunosuppressive TAMs, whereas the B16F10 model shows a lower abundance of TAMs but greater diversity of local immune cells. These differences contribute to divergent responses to immunotherapy between the two models (48). Thus, we compared the anti-tumor efficacy of ex vivo-derived DCs in these two models with distinct immune landscapes.

**Figure 6:**
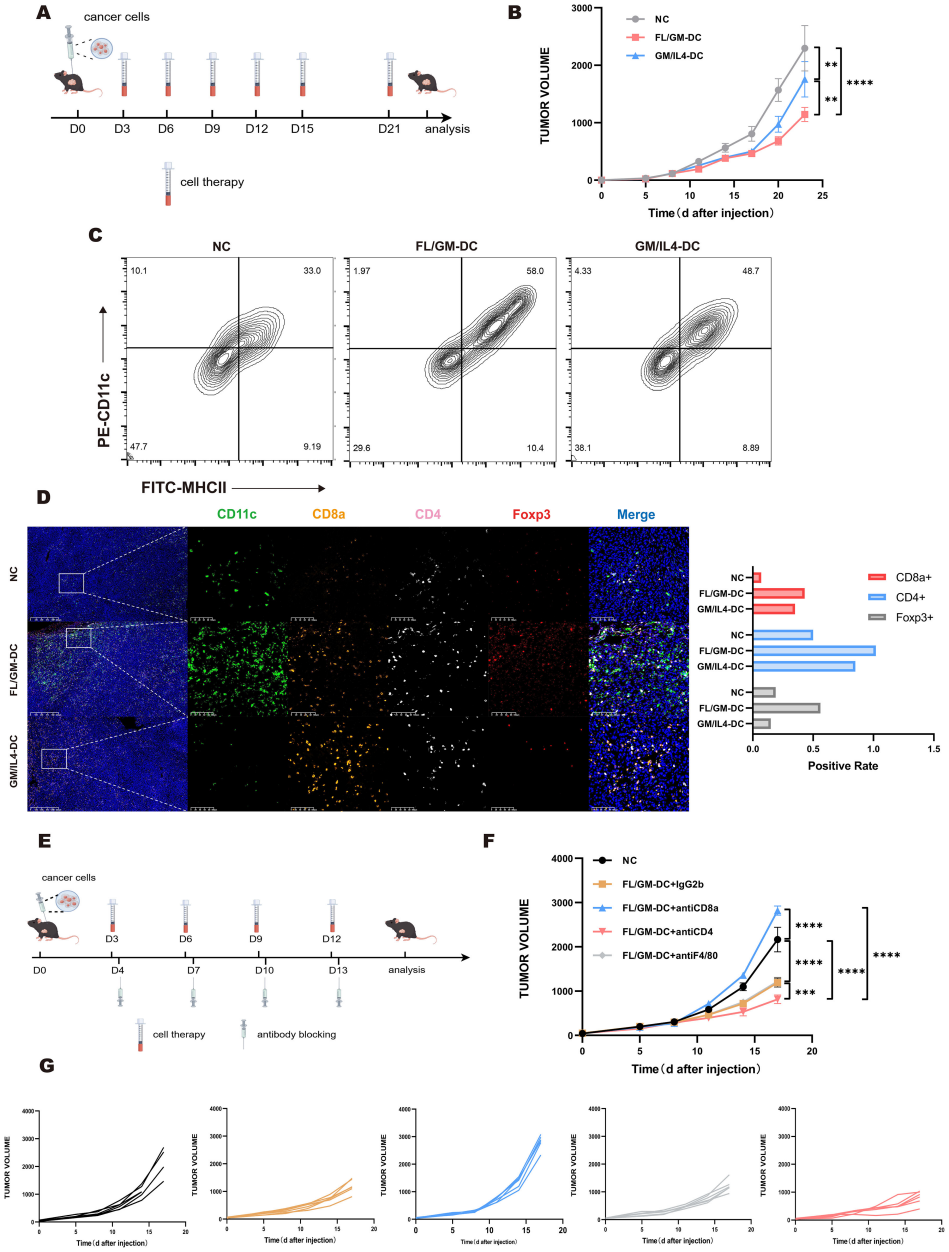
The anti-tumor effect of FL/GM-DC in tumor models. **(a)** Experimental protocol for studying the anti-tumor effects of FL/GM-DCs and GM/IL4-DCs in the tumor models. Mice with tumors were vaccinated on days 3, 6, 9, 12, 15, and 21 with FL/GM-DCs or GM/IL4-DCs (Each mouse received 2.5 x 10^6^ cells per administration). Measured the size of the tumor every 3 days. **(b)** Tumor size in MC38 tumor-bearing mice measured over time (mean ± SEM; NC, n=8; FL/GM-DC, n=8; GM/IL4-DC, n=8). Statistical analysis was performed using two-way ANOVA with Tukey’s multiple comparisons test. **(c)** Flow cytometric analysis of the proportion of infiltrating DCs (MHCII^+^CD11c^+^) in MC38 tumor tissue. **(d)** Representative fluorescence images of MC38 tumors from treated mice, showing CD11c (green), CD8a (orange), CD4 (pink), Foxp3 (red), and DAPI nuclear staining (blue). Scale bar, 100 μm. The bar chart represented the positive rate of CD8a^+^, CD4^+^, and Foxp3^+^ cells in the tumors of each group of MC38 mice. **(e)** Experimental research protocol on the anti-tumor effects of FL/GM-DCs and GM/IL4-DCs in an antibody-blockaded MC38 tumor model. Mice with tumors were vaccinated on days 3, 6, 9, 12 with FL/GM-DCs or GM/IL4-DCs (Each mouse received 2.5 x 106 cells per administration) and received antibody blocking therapy (Each mouse received 200ug antibodies per administration) on the next day. Measured the size of the tumor every 3 days. **(f)** Tumor size in MC38 tumor-bearing mice measured over time (mean ± SEM; NC, n=6; FL/GM-DC+IgG2b, n=6; FL/GM-DC+antiCD8a, n=6; FL/GM-DC+antiCD4, n=6; FL/GM-DC+antiF4/80, n=6). Statistical analysis was performed using two-way ANOVA with Tukey’s multiple comparisons test. **(g)** Each subplot displayed the tumor volume over time for individual mice in different treatment groups (NC, FL/GM-DC+IgG2b, FL/GM-DC+antiCD8a, FL/GM-DC+antiCD4, and FL/GM-DC+antiF4/80).

First, FL/GM-DCs or GM/IL4-DCs were administered via footpad injection in mice bearing subcutaneous B16F10 melanoma, using the protocol shown in **Figure 6a**. Compared with the negative control and GM/IL4-DC groups, FL/GM-DCs demonstrated an ability to inhibit tumor growth in the B16F10 model (**Supplementary Figure 4g**). GM/IL4-DCs failed to suppress tumor growth in this model. Despite the modest effect on tumor suppression, multiplex immunohistochemistry analysis of tumor tissues from the FL/GM-DC group revealed higher levels of immune cell infiltration, suggesting stronger immune activation by FL/GM-DCs (**Supplementary Figure 4h**). This finding implies that although FL/GM-DCs enhanced immune cell infiltration, the advantage did not lead to robust tumor growth inhibition. To further investigate immune cell infiltration, T cells within tumor tissues were quantified by flow cytometry. The results showed that tumors from the FL/GM-DC group contained a higher number of infiltrating T cells, predominantly CD8^+^ T cells. The analysis of regulatory T cells (Tregs, Foxp3^+^CD25^+^) revealed no significant differences between the groups (**Supplementary Figures 4i, j**).

MC38 tumor-bearing mice were treated according to the protocol used in the B16F10 study (**Figure 6a**). FL/GM-DCs achieved significantly better tumor control compared with GM/IL4-DCs (**Figure 6b**). Flow cytometric analysis of intra-tumoral immune cells revealed enhanced immune cell infiltration in the FL/GM-DC group. FL/GM-DCs substantially increased the number of MHCII^+^CD11c^+^ cells compared with GM/IL4-DCs (**Figure 6c**). The results of multiplex immunohistochemistry indicated that both FL/GM-DCs and GM/IL4-DCs increased CD8^+^ T cell and CD4^+^ T cell infiltration relative to the control group; however, FL/GM-DCs elicited a more pronounced effect (**Figure 6d**).

Based on these observations, we further examined the role of specific immune cell populations in the tumor microenvironment during adoptive DC therapy. Using monoclonal antibodies against F4/80, CD8a, and CD4, we selectively depleted TAMs, CD8^+^ T cells, and CD4^+^ T cells, respectively (**Figure 6e**). The results showed that the absence of CD8^+^ T cells substantially diminished the tumor-suppressive effect of FL/GM-DCs. Depleting TAMs did not affect the anti-tumor efficacy of FL/GM-DCs. Interestingly, depleting CD4^+^ T cells enhanced the anti-tumor efficacy of FL/GM-DCs, possibly due to the reduction in Tregs, thereby decreasing immunosuppression and potentiating the therapeutic effect against cancer (**Figures 6f, g**).

### FL/GM-DC therapy remodel immune microenvironment and induce CD8^+^ specific anti-tumor activity

**Figure 7:**
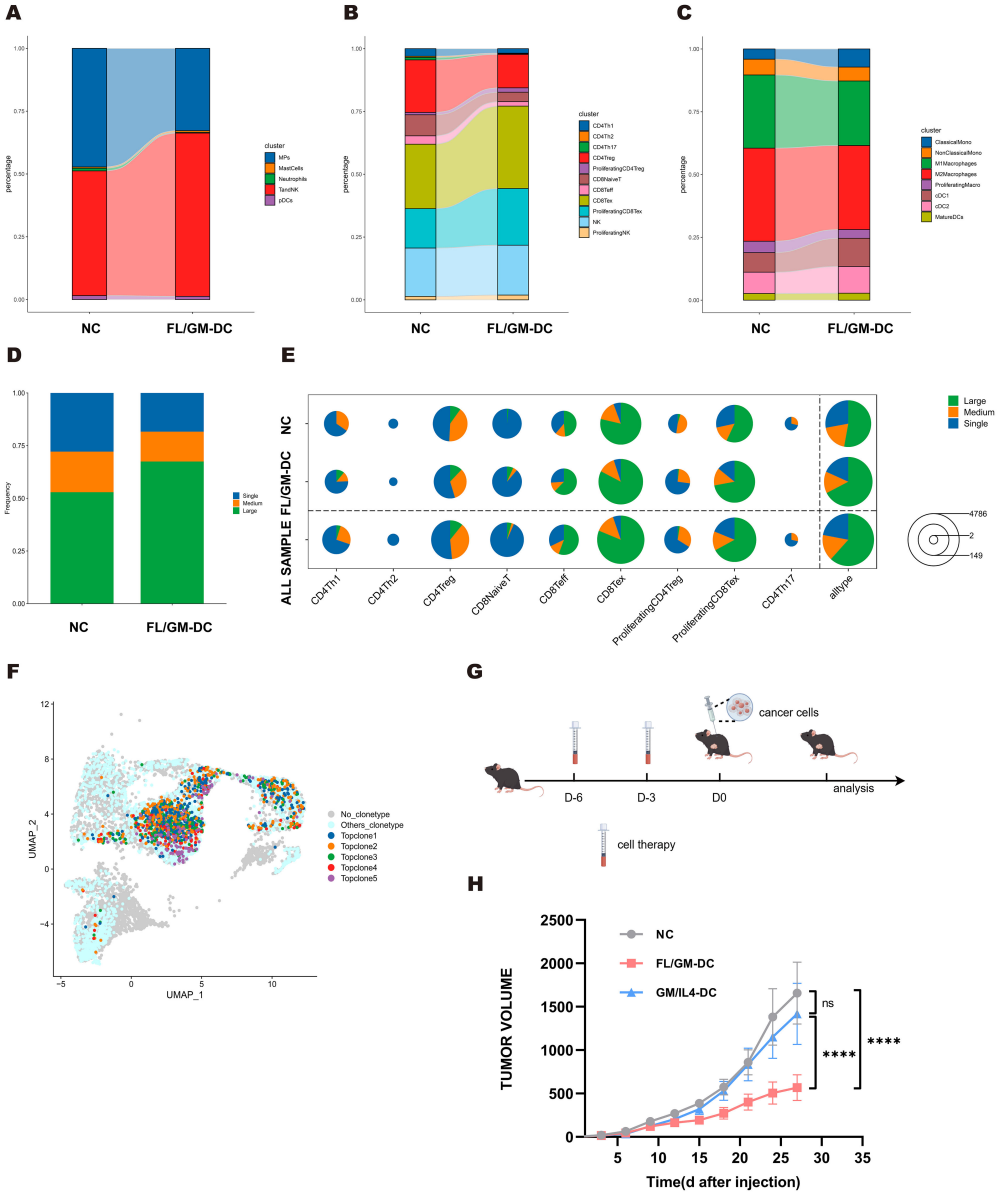
MC38 tumors exhibited improved tumor microenvironment following FL/GM-DCs treatment. **(a)** The stacked bar chart showed the percentage distribution of different immune cells in the NC group and the FL/GM-DC group, with each color representing a different type of immune cell. **(b)** The stacked bar chart showed the percentage distribution of different clusters of T and NK cells in the NC group and the FL/GM-DC group, with each color representing a different type of immune cell. **(c)** The stacked bar chart showed the percentage distribution of different clusters of MPs in the NC group and the FL/GM-DC group, with each color representing a different type of immune cell. **(d)** The stacked bar chart represented the proportions of TCR clones of different frequency in the NC and FL/GM-DC groups, where blue indicates single clones (Single), orange indicates medium-sized clones (Medium), and green indicates large clones (Large). **(e)** The pie chart illustrated the distribution of clone sizes for various T cell subpopulations (CD4Th1, CD4Th2, CD4Treg, CD8NaiveT, CD8Teff, CD8Tex, Proliferating CD4Treg, Proliferating CD8Tex, CD4Th17) in different sample groups (NC, FL/GM-DC, and ALL SAMPLE). Green represented large clones (Large), orange represented medium-sized clones (Medium), and blue represented single clones (Single). **(f)** Distribution of Top TCR clonotypes in UMAP. **(g)** Experimental protocol to assess the protective effects of FL/GM-DCs and GM/IL4-DCs against tumor challenge. On days -6 and -3, FL/GM-DCs or GM/IL4-DCs (Each mouse received 2.5 x 10^6^ cells per administration) were transferred into naïve mice, followed by subcutaneous inoculation of tumor cells on day 0. Measured the size of the tumor every 3 days. **(h)** Tumor sizes in MC38 tumor-bearing mice measured over time (mean ± SEM; NC, n=6; FL/GM-DC, n=6; GM/IL4-DC, n=6). Statistical analysis was performed using two-way ANOVA with Tukey’s multiple comparisons test.

To further elucidate how FL/GM-DC therapy suppresses tumor growth by reshaping immune cell subsets, abundance, and activation states within the tumor microenvironment, we harvested tumor tissues from both FL/GM-DC-treated and control mice at the conclusion of therapy in the MC38 model. We performed scRNA-seq and scTCR-seq of CD45^+^ immune cells isolated from both FL/GM-DC treated and control tumors. With canonical marker genes, we identified major immune cell population including T and NK, Monocytic phagocytes (MPs), Neutrophils, Mast cells and pDCs, which were further validated by mapping DEGs in each group (**Supplementary Figure 5a**). T and NK group and MPs group were subsequently extracted and reclustered separately. By mapping DEGs of each cluster, we identified distinct subsets including CD4 Th1, CD4 Th2, CD4 Th17, CD4 Treg, proliferating CD4 Treg, CD8 naïve T, CD8 T effector, CD8 T exhaustion (Tex), proliferating CD8 exhaustion (pTex), NK, proliferating NK in T and NK group (**Supplementary Figure 5b**). MPs group was classified into classical and nonclassical monocyte, M1/M2 macrophage, proliferating macrophage, cDC1/2 and mature DC (**Supplementary Figure 5c**). Further comparative analysis revealed significant alterations in immune cell proportions induced by FL/GM-DC therapy. Compared to the control group, FL/GM-DC treatment increased the proportion of T and NK cells and concomitantly reduced MPs infiltration (**Figure 7a**). Within the T cell subsets, the numbers of infiltrating Tex and pTex were notably elevated, whereas Tregs decreased (**Figure 7b**). Intriguingly, the proportion of CD4 Th cells, already low in control group, further decreased following FL/GM-DC therapy. The persistently high proportion of Tregs in CD4^+^ T cell compartment might explain why CD4 blockade resulted in slight suppression of tumor growth. Within the MP subsets, FL/GM-DC administration significantly decreased the infiltration of TAMs and elevated number of cDCs. Additionally, the ratio of M1/M2 remained balance in TME following FL/GM-DC therapy, explaining why F4/80 blockade show no significant effect on tumor growth (**Figure 7c**). These data collectively indicate that FL/GM-DC therapy promotes an immune-activating microenvironment characterized by enhanced effector T cell activity and reduced immunosuppression, thereby contributing to improved anti-tumor immunity.

Analysis of TCR-seq further demonstrated that FL/GM-DC treatment induced robust antigen-specific T cell responses within the TME. Specifically, FL/GM-DC transfer significantly increased the proportion of highly expanded TCR clones (from 52.9% to 67.5%), accompanied by a reduction in low- and medium-frequency clones (**Figure 7d**), overall TCR diversity—as assessed by Hill numbers and the Inverse Simpson index—was decreased following FL/GM-DC treatment (**Supplementary Figures 5d, e**), suggesting the emergence of potent, tumor-specific T cell reactivity. Detailed analyses showed that these expanded high-frequency TCR clones mapped to all CD8^+^ clusters (**Figure 7e**). Additionally, TCR clonotype overlap analysis revealed enhanced sharing of clonotypes between Tex and pTex subsets (increased from 37 to 60 clonotypes) (**Supplementary Figures 5f, g**), indicating active clonal expansion within CD8^+^ T cell subsets induced by FL/GM-DCs. The total numbers of distinct TCR clones increased among the Tex and pTex subsets, indicating that FL/GM-DC therapy elicited CD8^+^ T cell responses against a broader range of tumor antigens. Finally, we identified top five most abundant TCR clones from both groups and mapped onto the UMAP plot. Notably, four clonotypes were originated from FL/GM-DC group and were largely restricted to Tex and pTex clusters (**Figure 7f**). Collectively, these data strongly support the conclusion that FL/GM-DC adoptive transfer stimulates robust, antigen-specific CD8^+^ T cell responses in the TME, thereby facilitating enhanced anti-tumor immunity.

### Vaccination with FL/GM-DCs protects against tumor challenges

Considering that DCs activate adaptive immune responses and generate immune memory, adoptive DC therapy is often utilized as a tumor vaccine. To evaluate the efficacies of FL/GM-DCs and GM/IL4-DCs as tumor vaccines, naïve mice were inoculated via footpad with either antigen loaded FL/GM-DCs or GM/IL4-DCs 6 days before tumor challenging a booster vaccination was given 3 days later. Then mice were challenged with subcutaneous inoculation of B16F10 melanoma cells or MC38 colorectal carcinoma cells (**Figure 7g**). Mice vaccinated with FL/GM-DCs exhibited a substantial reduction in tumor growth relative to the control group and GM/IL4-DC-vaccinated mice (**Figure 7h**, and **Supplementary Figure 5h**). Multiplex immunohistochemistry analysis of B16F10 tumor tissues revealed extensive infiltration of DCs, CD8^+^ T cells, and CD4^+^ T cells in the tumor microenvironment of FL/GM-DC-vaccinated mice (**Supplementary Figure 5i**). These results indicate that ex vivo-derived bone marrow DCs induce durable and robust anti-tumor effects.

## Discussion

cDCs play a pivotal role in orchestrating anti-tumor immunity (49). In many solid tumors, a higher abundance of cDCs, particularly cDC1s, is positively correlated with favorable patient outcomes, underscoring their potential in adoptive cell therapy (50). However, the limited availability of cDCs *in vivo* remains a significant challenge for clinical applications, as efficient enrichment and expansion of these cells for therapeutic use remain technically challenging (51). The FLT3L-FLT3 receptor signaling pathway is a key regulator of DC development, particularly for cDCs (52, 53). Previous studies have demonstrated that hematopoietic stem and progenitor cells (HSPCs) can be differentiated into DC populations that closely resemble their steady-state counterparts *in vivo* using an FLT3L-based culture system, providing a robust platform for studying DC biology (54). This approach also offers a promising strategy for generating high-purity cDCs, for potential use in adoptive cell-based immunotherapies.

In our study, we first established an FLT3L/GMCSF-based culture system supplemented with low-dose GM-CSF to generate FL/GM-DCs for tumor therapy. Flow cytometry analysis of surface markers confirmed that FL/GM-DCs exhibited a higher proportion of cDC, including cDC1 and cDC2, compared to GM/IL4-DCs. To enhance the identification of DC subsets, we optimized a panel that effectively distinguishes DC populations. Initially, CD88 and SiglecH were used to differentiate monocyte-derived cells and pDCs. Next, CCR7 and CCR5 were employed to distinguish mature and immature DCs, followed by further subdivision into cDC1 and cDC2 subsets based on XCR1, CLEC9a, DCIR2, and CD172a expression. Interestingly, we observed that XCR1 and DCIR2 were expressed at the progenitor cell stage, preceding the upregulation of CLEC9a and CD172a. Given that CLEC9a and CD172a are both associated with antigen presentation, their delayed expression suggests a role in functional maturation. These findings indicate that XCR1 and DCIR2 may serve as useful markers for distinguishing progenitor DC subsets prior to full maturation.

Although previous studies have established that GM/IL4-DCs comprise a heterogeneous population, including macrophages and bona fide DCs, a comprehensive characterization of their DC subtypes, functional states, and differences from FL/GM-DCs remains lacking. In this study, we systematically analyzed and compared the cellular composition, proportions, and functional characteristics of FL/GM-DCs and GM/IL4-DCs. It is important to note that for downstream functional assays and *in vivo* experiments, we utilized the entire cell population induced by each culture method (FLT3L/GM-CSF or GM-CSF/IL-4) without further purification. We acknowledge that this approach, using bulk cells, has the inherent limitation of potentially including non-DC cells or multiple DC subsets, which could introduce background noise. Our findings revealed that while both FL/GM-DCs and GM/IL4-DCs contained a high proportion of mature DCs, they exhibited substantial functional differences. Specifically, mature DCs from FL/GM-DCs demonstrated superior capacity for activating T-cell immune responses, whereas mature DCs from GM/IL4-DCs displayed a phenotype more closely resembling monocyte-derived macrophages. Furthermore, aside from mature DCs, cDC and pDC subsets were largely absent in GM/IL4-DCs, which is consistent with our flow cytometry data. This skewed differentiation limits the generation of a complete spectrum of DC subtypes, thereby restricting the full potential of DC-based anti-tumor immunity.

Additionally, we mapped the cDC subclusters within FL/GM-DCs, providing a detailed atlas of their heterogeneity. Within FL/GM-DCs, we identified a homogeneous cDC1 population and a highly heterogeneous cDC2 population. DEG analysis revealed distinct functional and cellular states among cDC2 subsets, including ISG^+^DCs, transitional DCs (tDCs), moDC-like cDC2, and potentially tolerogenic cDC2. Previous studies have described ISG^+^ DCs as an alternative maturation state distinct from CCR7^+^ mature DCs, in which IFN signaling plays a dominant role (39). Duong et al. demonstrated that ISG^+^ DCs can activate CD8^+^ T cells through MHC class I dressing, thereby enhancing anti-tumor responses (37). In FL/GM-DC-derived cDC2 subsets, Cluster 2 exhibited key ISG^+^ DC features, including Ly6a, Fcgr1, and Axl. Interestingly, we also identified the specific upregulation of Apol7c in Cluster 2, a pore-forming apolipoprotein known to promote cross-presentation via phagosomal rupture. This finding suggests that Apol7c may be one of the potential mechanisms by which ISG^+^ DCs enhance CD8^+^ T cell activation. However, this hypothesis requires further validation through functional experiments. Although ISG^+^ DCs have traditionally been considered a subset of cDC2 (CD11b^+^ cDCs), our data indicate that cDC1s within FL/GM-DCs also exhibit partial ISG^+^ DC gene expression signatures. This observation suggests that the IFN-driven maturation pathway distinct from CCR7^+^ DC maturation may occur in both cDC1 and cDC2 subsets. This finding has important implications for DC-based immunotherapies, as mature ISG^+^ DCs within TME may retain antigen-presenting capacity while simultaneously recruiting T cells via CXCL9/10 and directly priming them *in situ*. By bypassing the requirement for CCR7-mediated lymph node homing and subsequent T cell activation, ISG^+^ DCs could accelerate antigen-specific anti-tumor responses and promote sustained DC-T cell interactions within the TME. This novel mechanism warrants further investigation. Furthermore, our bioinformatic analyses predicted that certain cDC2 clusters may possess either tolerogenic or immunostimulatory functions based on their gene expression profiles. However, these predictions remain to be experimentally validated. Future studies employing functional assays, such as T cell suppression or activation assays, as well as *in vivo* models targeting specific cDC2 subsets, will be essential to confirm these putative roles and to elucidate the underlying mechanisms by which these subsets modulate immune responses within the tumor microenvironment.

Consistent with our *in vitro* findings, FL/GM-DCs exhibited significantly greater therapeutic efficacy than GM/IL4-DCs in both B16F10 melanoma and MC38 colorectal cancer models, whether applied as a tumor vaccine or as a therapeutic intervention after tumor onset. These results further support the potential application of FL/GM-DCs as a novel therapeutic strategy for solid tumors. *In vivo* analyses, including immune cell subset-specific blockade, multiplex immunohistochemistry of tumor tissues, and scRNA-seq of tumor-infiltrating immune cells, were in line with our *in vitro* observations, demonstrating that FL/GM-DCs were highly proficient in activating and expanding CD8^+^ T cells. Furthermore, TCR-seq analysis confirmed that FL/GM-DCs induced a broad and antigen-specific CD8^+^ T cell response *in vivo*, providing direct evidence of their role in remodeling the tumor microenvironment (TME) and driving anti-tumor immunity. Following FL/GM-DC therapy, a substantial expansion of Tex and pTex was observed, accompanied by upregulation of immune checkpoint molecules such as *Pdcd1* and *Lag3*. These findings suggest that combining FL/GM-DC therapy with immune checkpoint inhibitors may prolong the anti-tumor activity of CD8^+^ T cells, further enhancing therapeutic efficacy.

The B16F10 melanoma model, typically considered less immunogenic than the MC38 colorectal cancer model, showed differences in treatment response (48). Both FL/GM-DCs and GM/IL4-DCs were more effective in the MC38 model, whereas GM/IL4-DCs failed to inhibit tumor growth in the B16F10 melanoma model. Intriguingly, when induced DCs were used as a tumor vaccine, their therapeutic effect was significantly greater in the B16F10 melanoma model than in the MC38 model. This outcome contrasts with the results observed when treatment was administered after tumor establishment. Considering the higher content of TAMs in MC38 tumors compared with B16F10 tumors, we speculate that—when used therapeutically—the injected DCs activate T cells to initiate an anti-tumor response while exerting tumoricidal effects through the accumulation of CD11c^+^ cells. These accumulated cells include M1-polarized macrophages and differentiated moDCs, which may secrete TNF-α and reactive oxygen species to directly kill tumor cells. In the context of tumor vaccination, the absence of established tumor lesions means that this macrophage-mediated effect does not occur. In contrast, B16F10 tumors have a lower TAM content and primarily rely on T cell-mediated anti-tumor immunity, likely explaining the therapeutic advantage observed in this model and highlighting divergent therapeutic outcomes between the two models.

Overall, this study has established the feasibility of adoptive transfer of FL/GM-DCs as a strategy for cancer immunotherapy. However, several key issues remain to be elucidated. A central point is that the DC population generated by the current method exhibits significant heterogeneity, encompassing cDCs, pDCs, and mature DCs. While we have conducted a thorough analysis of the cDC2 subset, the observed heterogeneity within mature DCs is also noteworthy, suggesting that DCs at different maturation stages may play diverse roles in anti-tumor immunity. Therefore, deeply investigating the functional diversity among different DC subsets and their specific contributions to the tumor immune response is a top priority for future research. Although our existing proteomic and transcriptomic data provide valuable insights into the functional states of DC subsets, experimentally validating this by isolating and functionally characterizing individual subsets both *in vitro* and *in vivo* is crucial. Next, utilizing high-precision flow cytometry to meticulously sort the heterogeneous DC subsets generated by FL/GM-DC induction based on cell surface markers, followed by functional testing of each isolated subset, is particularly critical for the successful translation of the FL/GM-DC therapy into clinical application. Regarding the role of cDC1 in enhanced therapeutic efficacy, our study has not directly tested whether the observed improvements are specifically attributable to the enrichment of cDC1 cells. While our current data imply a potential contribution of cDC1 to the overall anti-tumor response, direct experimental evidence establishing causality is still lacking. To address this, future studies should employ targeted approaches such as cDC1 depletion models (e.g., XCR1-DTR mice) or selective cDC1 blockade to definitively determine whether cDC1 enrichment is a key driver of therapeutic efficacy. Such experiments are essential for validating the mechanistic role of cDC1 and optimizing DC-based immunotherapy strategies.

Finally, it is important to recognize that clinical protocols for GM/IL4-DCs typically utilize peripheral blood CD14^+^ monocytes as the starting material (55), whereas our current study employs bone marrow cells. Although BM contains a proportion of monocytes, it also includes a diverse pool of progenitor cells with greater differentiation potential. While GM-CSF induction of BM cells can generate mature DCs, GM-CSF treatment of fully differentiated cells (e.g., CD14^+^ monocytes) primarily yields monocyte-derived cells (Macrophages/moDCs). Therefore, before advancing FL/GM-DC therapy to clinical applications, further validation using human cells is necessary to determine whether FLT3L/GM-CSF-induced differentiation of DCs from HSPCs provides substantial advantages over conventional GM-CSF/IL4 induction from peripheral blood CD14^+^ monocytes.

## Data Availability

The data presented in the study are deposited in the National Center for Biotechnology Information (NCBI) repository, accession number PRJNA1311986.
