# Supplementary material for: FLT3L combined with GM-CSF induced dendritic cells drive broad tumor-specific CD8+ T cell responses and remodel the tumor microenvironment to enhance anti-tumor efficacy
Source: Front Immunol. 2025 Sep 4;16:1649891. doi: 10.3389/fimmu.2025.1649891 (PMC12443778; doi:10.3389/fimmu.2025.1649891)
Supplement: Supplementary file 1 [file DataSheet1.docx]

***Supplementary Material***

**Supplementary Figure 1**


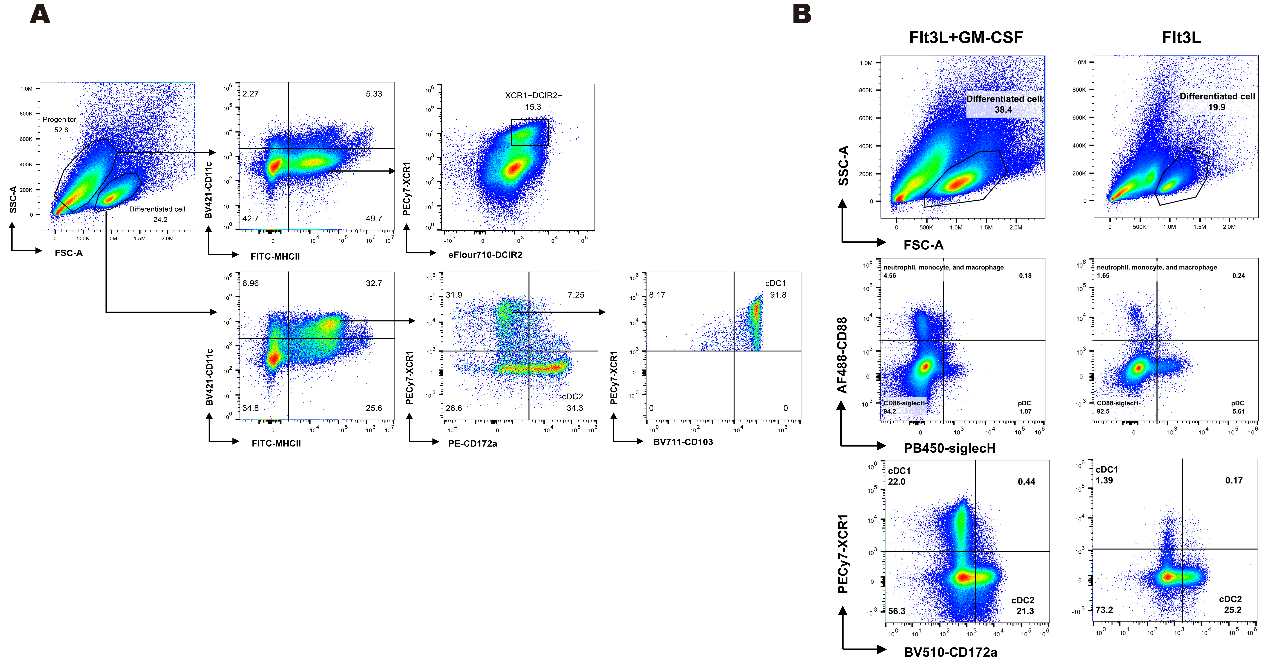


**Supplementary Figure 1: Cell Subpopulation Proportions in FLT3L and GM-CSF Culture System. a** Flow cytometry for analyzing the proportion of various cell subpopulations in the 100 ng/ml FLT3L+ 10 ng/ml GM-CSF culture system. **b** Flow cytometry analysis of comparing the proportions of various cell subpopulations under two different conditions: 100 ng/ml FLT3L+ 10 ng/ml GM-CSF and 100ng/ml FLT3L alone

**Supplementary Figure 2**


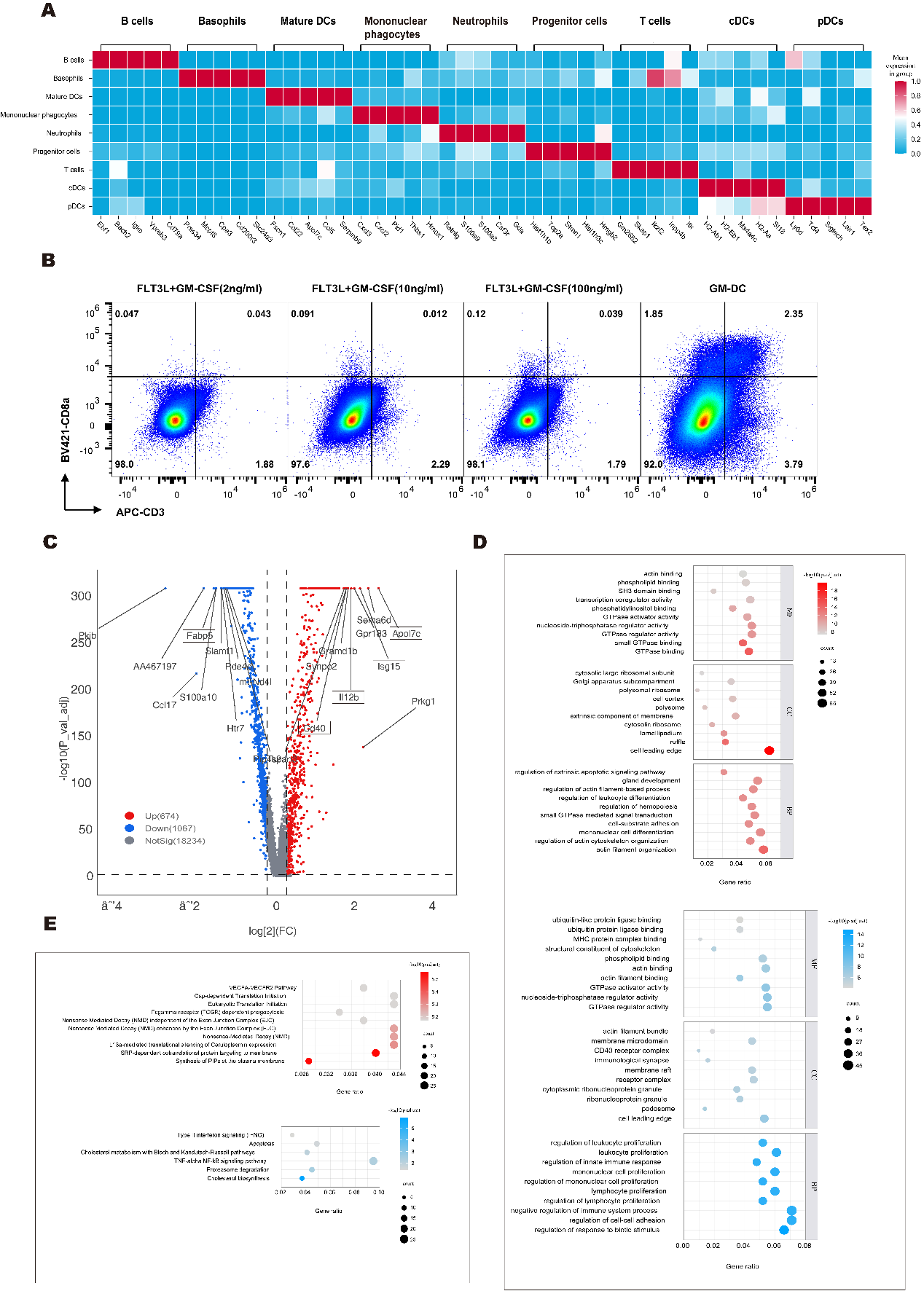


**Supplementary Figure 2: Characterization of DC Subpopulations Derived from FL/GM-DC Cultures. a** Heatmap of the top 5 marker genes for each cluster; blue indicated low expression and red indicated high expression. **b** Flow cytometry demonstrated the proportions of CD3^+^CD8a^+^ T cells in the FLT3L+GM-CSF (2ng/ml), FLT3L+GM-CSF (10ng/ml), FLT3L+GM-CSF (100ng/ml), and GM/IL4-DC groups. **c** Volcano plot of differentially expressed genes (DEG) between mature DC subpopulations derived from FL/GM-DC and GM/IL4-DC. The x-axis represented the logarithmic fold change of gene expression (log2(FC)), and the y-axis represented the significance of gene expression (-log10(P value)). **d** GO analysis of FL/GM-DCs in the mature DC subpopulation compared with GM/IL4-DCs. The vertical axis represented the enriched pathways, and the horizontal axis represents the ratio of the number of differentially expressed genes annotated to the GO terms to the total number of differentially expressed genes; the size of the circles in the graph indicated the number of genes, and the color gradient from red to blue represented the enrichment significance from high to low. **e** Wikipathway enrichment analysis of FL/GM-DCs in the mature DC subpopulation compared with GM/IL4-DCs

**Supplementary Figure 3**


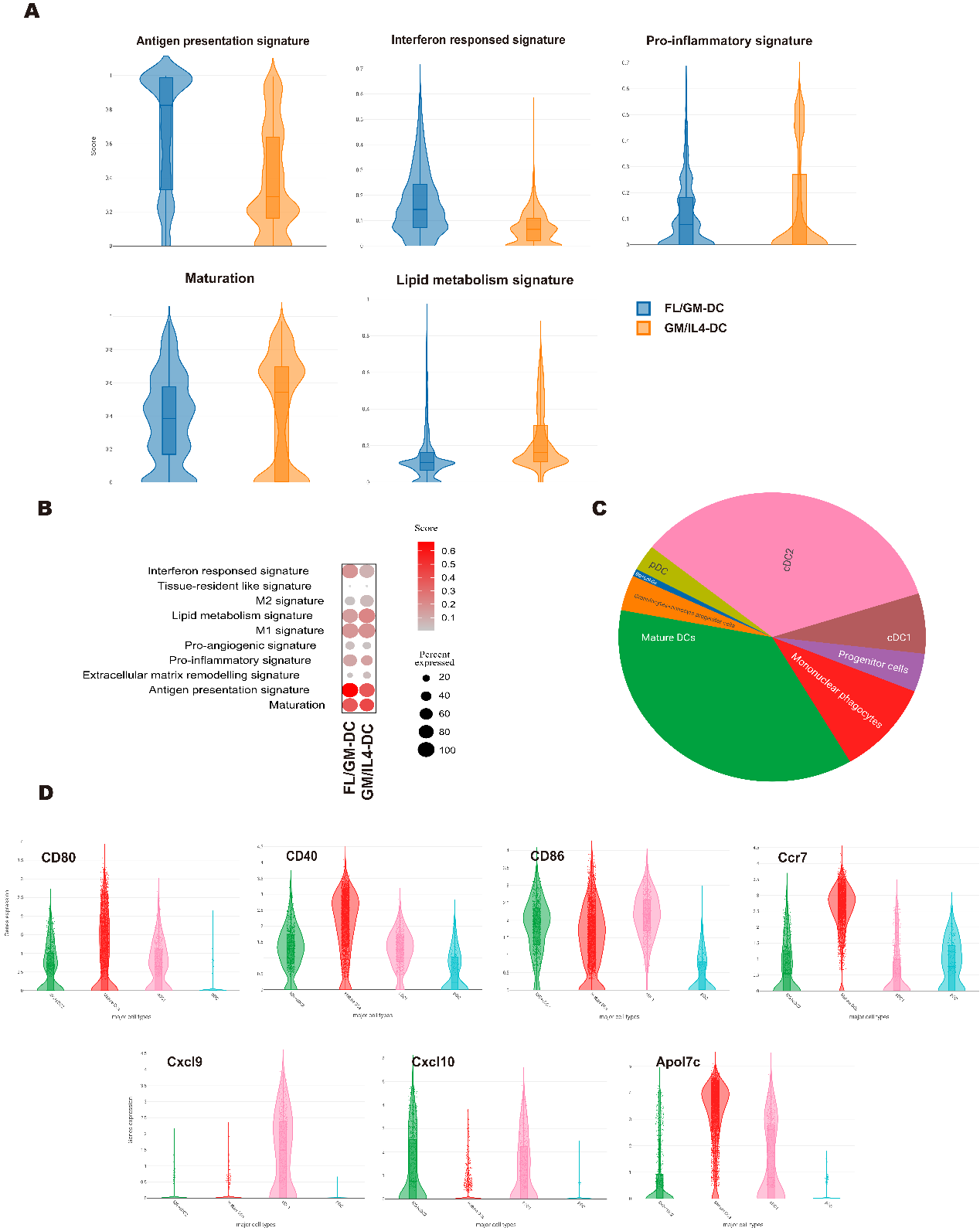


**Supplementary Figure 3: Gene Signatures and Cluster Distribution in Mature DCs from Different Culture Conditions. a, b** Violin plot and bubble chart displayed the Ucell-based gene set scores to evaluate the characteristics of different gene signatures in mature DCs within FL/GM-DC group and GM/IL4-DC group. **c** Pie chart showed the proportion of each cluster in the FL/GM-DC group. **d** Violin plot displayed the Ucell-based gene set score, to assess the expression levels of different maturation genes and T cell chemokines in various DC subpopulations within the FL/GM-DC group.

**Supplementary Figure 4**


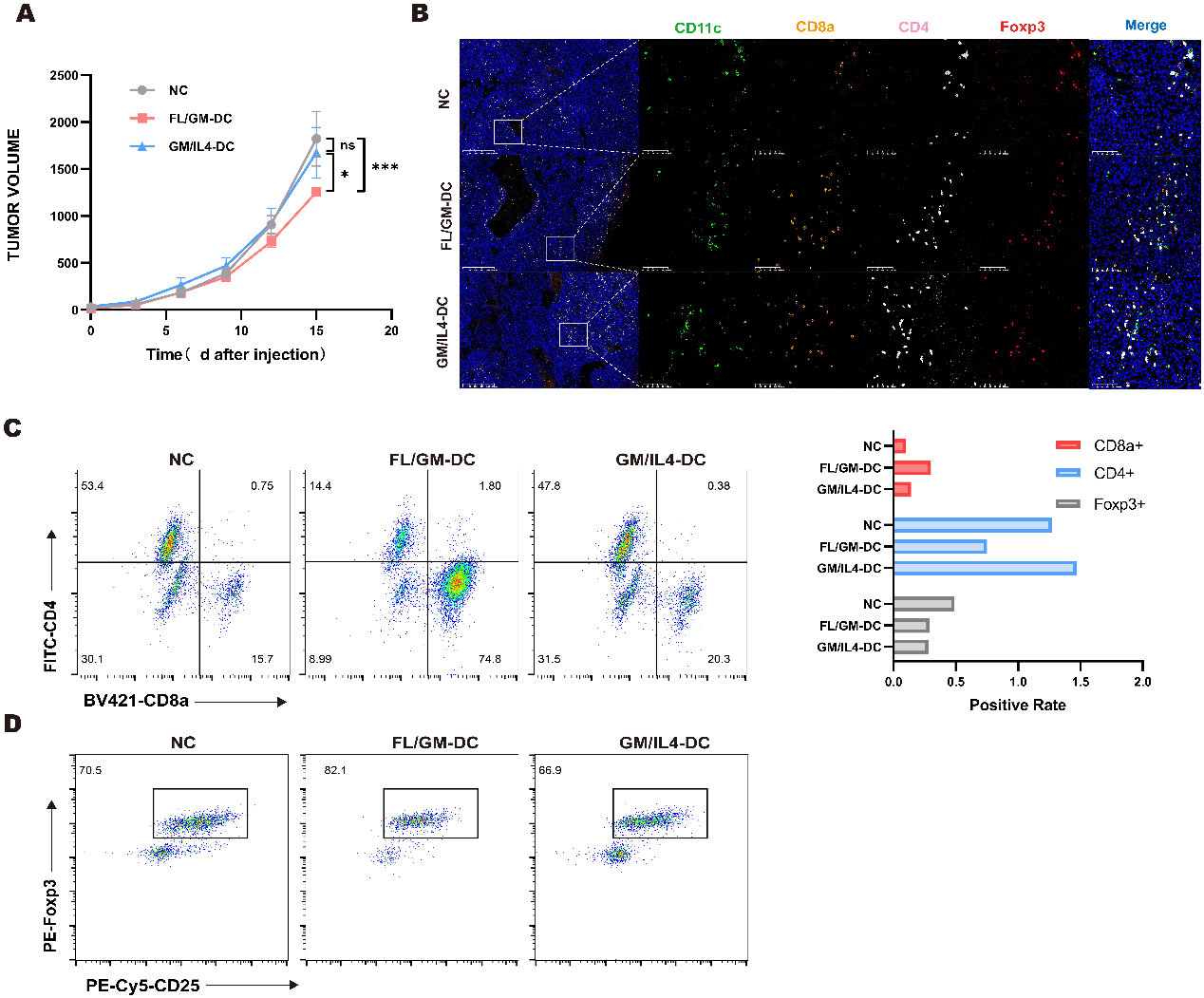


**Supplementary Figure 4: The anti-tumor effect of FL/GM-DC in other tumor models. a** Mean Fluorescence Intensity (MFI) of Ovalbumin (OVA) uptake by different cell populations. *p < 0.05, **p < 0.01, ***p < 0.001, ****p < 0.0001. **b** The level of antigen presentation was evaluated by measuring the mean fluorescence intensity (MFI) of H2Kb-SIINFEKL complexes on the FL/GM-DCs surface via flow cytometry. *p < 0.05, **p < 0.01, ***p < 0.001, ****p < 0.0001. **c** The level of antigen presentation was evaluated by measuring the MFI of H2Kb-SIINFEKL complexes on the GM/IL4-DCs surface via flow cytometry. *p < 0.05, **p < 0.01, ***p < 0.001, ****p < 0.0001. **d-f** Flow cytometry analysis of the MFI of CCR7 (d), CD103 (e), and CD86 (f) on MHCII^+^CD11c^+^cells in FL/GM-DCs and GM/IL4-DCs. **g** Gating strategy for flow cytometry analysis of adoptively transferred CD45.1^+^ cells. **h** Tumor size in B16F10 tumor-bearing mice measured over time (mean ± SEM; NC, n=8; FL/GM-DC, n=8; GM/IL4-DC, n=8). Statistical analysis was performed using two-way ANOVA with Tukey’s multiple comparisons test. **i** Representative fluorescence images of B16F10 tumors from treated mice, showing CD11c (green), CD8a (orange), CD4 (pink), Foxp3 (red), and DAPI nuclear staining (blue). Scale bar, 100 μm. The bar chart represented the positive rate of CD8a^+^, CD4^+^, and Foxp3^+^ cells in the tumors of each group of B16F10 mice. **j** Flow cytometric analysis of infiltrating CD8a^+^ and CD4^+^ T cells in tumor tissue. **k** Flow cytometric analysis showed the proportion of Treg cells (CD25^+^Foxp3^+^) within the CD4^+^ T cell population

**Supplementary Figure 5**


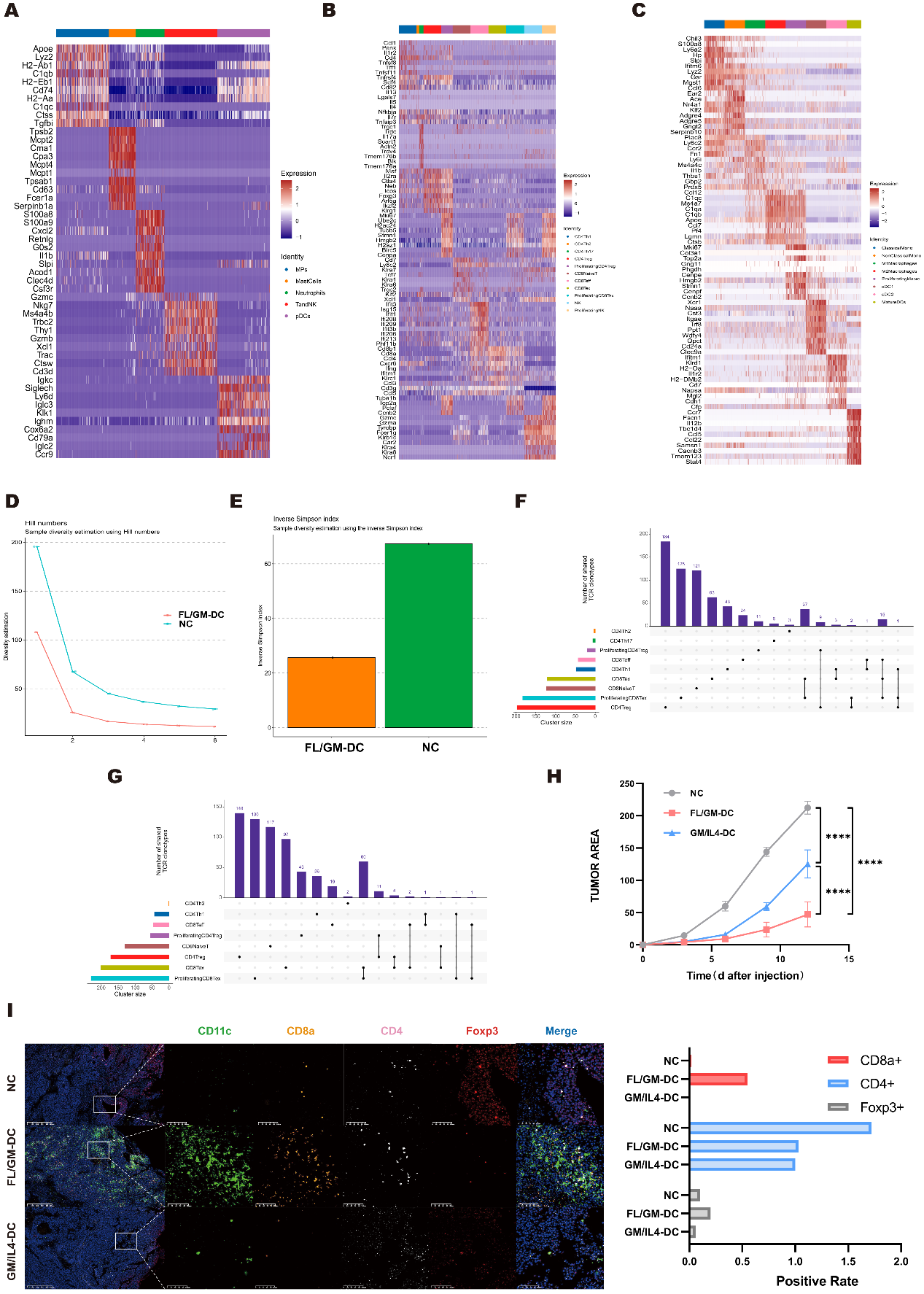


**Supplementary Figure 5: a** In the MC38 model, tumor tissues were collected from both FL/GM-DC-treated mice and control mice at the conclusion of therapy. ScRNA-seq was performed on CD45^+^ immune cells both the FL/GM-DC treatment group and the control group. Heatmap identified the DEGs of the major immune cell populations. Purple indicated low expression and red indicated high expression. **b** After re-clustering, the DEGs for each cluster of T and NK cells were determined through a heatmap. **c** The DEGs for each cluster of MPs were determined through a heatmap. **d** The line chart displayed the diversity estimates for the NC and FL/GM-DC groups calculated using the Hill numbers method. The x-axis represents the exponent q of the Hill number, and the y-axis represents the diversity estimate. **e** The bar graph illustrated sample diversity estimation using the Inverse Simpson Index for two groups: FL/GM-DC and NC. The height of each bar represented the Inverse Simpson Index value. **f, g** The distribution of the number of shared TCR clones in different T cell subpopulations in the NC group (f) and FL/GM-DC (g). **h** Tumor sizes in B16F10 tumor-bearing mice measured over time (mean ± SEM; NC, n=6; FL/GM-DC, n=6; GM/IL4-DC, n=6). Statistical analysis was performed using two-way ANOVA with Tukey’s multiple comparisons test. **i** Representative fluorescence images of B16F10 tumors from treated mice, showing CD11c (green), CD8a (orange), CD4 (pink), Foxp3 (red), and DAPI nuclear staining (blue). Scale bar, 100 μm. The bar chart represented the positive rate of CD8a^+^, CD4^+^, and Foxp3^+^ cells in the tumors of each group of B16F10 mice.
